# Fibroblast growth factor receptor signaling plays a key role in transformation induced by the TMPRSS2/ERG fusion gene and decreased PTEN

**DOI:** 10.18632/oncotarget.24470

**Published:** 2018-02-12

**Authors:** Longjiang Shao, Jianghua Wang, Omer Faruk Karatas, Shu Feng, Yiqun Zhang, Chad J. Creighton, Michael Ittmann

**Affiliations:** ^1^ Deptartment of Pathology & Immunology, Baylor College of Medicine, Houston, Texas 77030, USA; ^2^ Michael E. DeBakey Department of Veterans Affairs Medical Center, Houston, Texas 77030, USA; ^3^ Department of Medicine, Baylor College of Medicine, Houston, Texas 77030, USA; ^4^ Dan L. Duncan Cancer Center Division of Biostatistics, Baylor College of Medicine, Houston, Texas 77030, USA

**Keywords:** prostate cancer, signal transduction, fibroblast growth factors, PTEN, TMPRSS2/ERG

## Abstract

Prostate cancer is the most common visceral malignancy and the second leading cause of cancer deaths in US men. Correlative studies in human prostate cancers reveal a frequent association of the TMPRSS2/ERG (TE) fusion gene with loss of PTEN and studies in mouse models reveal that ERG expression and PTEN loss synergistically promote prostate cancer progression. To determine the mechanism by which ERG overexpression and PTEN loss leads to transformation, we overexpressed the TE fusion gene and knocked down PTEN in an immortalized but non-transformed prostate epithelial cell line. We show that ERG overexpression in combination with PTEN loss can transform these immortalized but non-tumorigenic cells, while either alteration alone was not sufficient to fully transform these cells. Expression microarray analysis revealed extensive changes in gene expression in cells expressing the TE fusion with loss of PTEN. Among these gene expression changes was increased expression of multiple FGF ligands and receptors. We show that activation of fibroblast growth factor receptor signaling plays a key role in transformation induced by TE fusion gene expression in association with PTEN loss. In addition, *in vitro* and in silico analysis reveals PTEN loss is associated with widespread increases in FGF ligands and receptors in prostate cancer. Inhibitors of FGF receptor signaling are currently entering the clinic and our results suggests that FGF receptor signaling is a therapeutic target in cancers with TE fusion gene expression and PTEN loss.

## INTRODUCTION

Prostate cancer (PCa) is the most common malignancy in American men, affecting one in nine men over 65 years of age [1]. Currently there is no effective cure for the advanced stages of PCa and it is the second-leading cause of male cancer mortality. The TMPRSS2/ERG (TE) fusion gene is the most common genomic alteration in PCa and is present in approximately 50% of cases [2, 3]. This fusion results in high levels of ERG expression under the androgen regulated TMPRSS2 promoter. PCas with the TE fusion have been shown to have activation of multiple proteins and pathways such as Wnts, Sox 9, Ezh2, MYC, TGF-β and others [4–13]. We have shown that the TE fusion gene increases NF-κB mediated transcription via increased phosphorylation of NF-κB p65 on Ser536 [14] and this activation promotes tumorigenesis [15].

Correlative studies in human PCa reveal a frequent association of the TE fusion gene with loss of PTEN and studies in mouse models reveal that ERG expression and PTEN loss synergistically promote PCa progression [16–18]. Consistent with this observation, we have shown that ERG protein levels are positively associated with levels of phosphorylated AKT (Ser473) and phosphorylated GSK3β in PCa tissues [19]. The mechanistic basis for the association of ERG expression and PTEN loss is not clear. It has been shown that ERG restores decreased androgen receptor mediated transcription induced by PTEN loss and it has been suggested that this explains the cooperative ability of ERG and PTEN to induce transformation [20].

Fibroblast growth factors (FGFs) are a family of 18 different ligands that bind with variable affinity to four different FGF receptors (FGFR1-4)[21]. There is an extensive literature implicating increased FGFR signaling in the initiation and progression of PCa and the majority of both primary and metastatic cancers have increased expression of one or more FGF ligands and/or FGF receptors [21–31]. The underlying molecular alteration(s) driving this increased expression remain obscure.

To determine the mechanism by which TE fusion gene expression and PTEN loss leads to transformation, we expressed The TE fusion gene and/or knocked down PTEN in an immortalized but non-transformed prostate epithelial cell line. We show that TE expression in combination with PTEN loss can transform these immortalized but non-tumorigenic cells while either alteration alone was not sufficient to fully transform these cells. Furthermore, we show that activation of fibroblast growth factor receptor (FGFR) signaling plays a key role in transformation induced by ERG expression in association with PTEN loss. In addition, we show that PTEN loss results in widespread but variable increases in FGF ligands and receptors in PCa.

## RESULTS

### TE fusion gene expression and PTEN knockdown cooperate to transform prostate epithelial cells

**Figure 1:**
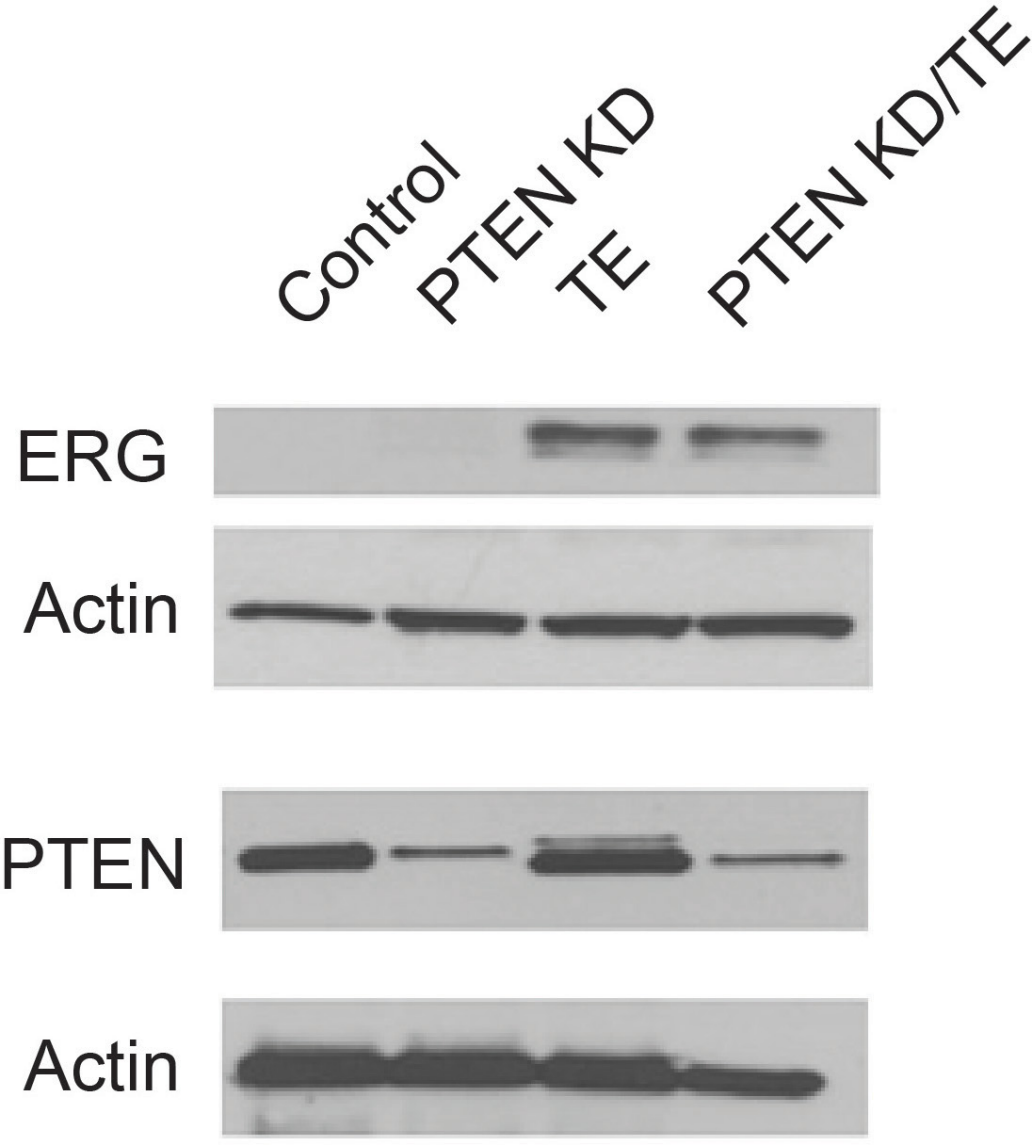
Characterization of protein expression in immortalized prostate cell lines with PTEN knockdown, TMPRSS2/ERG fusion gene expression or both alterations Stable cell lines were established from PNT1A immortalized human prostate epithelial cells with knockdown of PTEN (PTEN KD), overexpression of the TMPRSS2/ERG fusion gene (TE) or with both PTEN knockdown and fusion gene overexpression (PTEN KD/TE). Western blots for the TRMPRSS2/ERG fusion gene protein (ERG) and PTEN are shown along with β-actin loading controls.

To determine if TE fusion gene expression and PTEN loss were sufficient to transform prostate epithelial cells we constructed cell lines with expression of the TMPRSS2/ERG fusion gene (TE), stable knockdown of PTEN with shRNA (PTEN KD) or both alterations (PTEN KD/TE) using the PNT1A cell line. The PNT1A cell line was originally established by SV40 T-Ag immortalization of benign prostate epithelial cells [32]. PNT1A are immortal but do not form colonies in soft agar or tumors in immunocompromised mice. They do not express androgen receptor (AR), as is typical for benign prostate epithelial cells in culture. Western blotting confirms expression of the ERG fusion gene and knockdown of PTEN in the appropriate cell lines (Figure 1).

**Figure 2:**
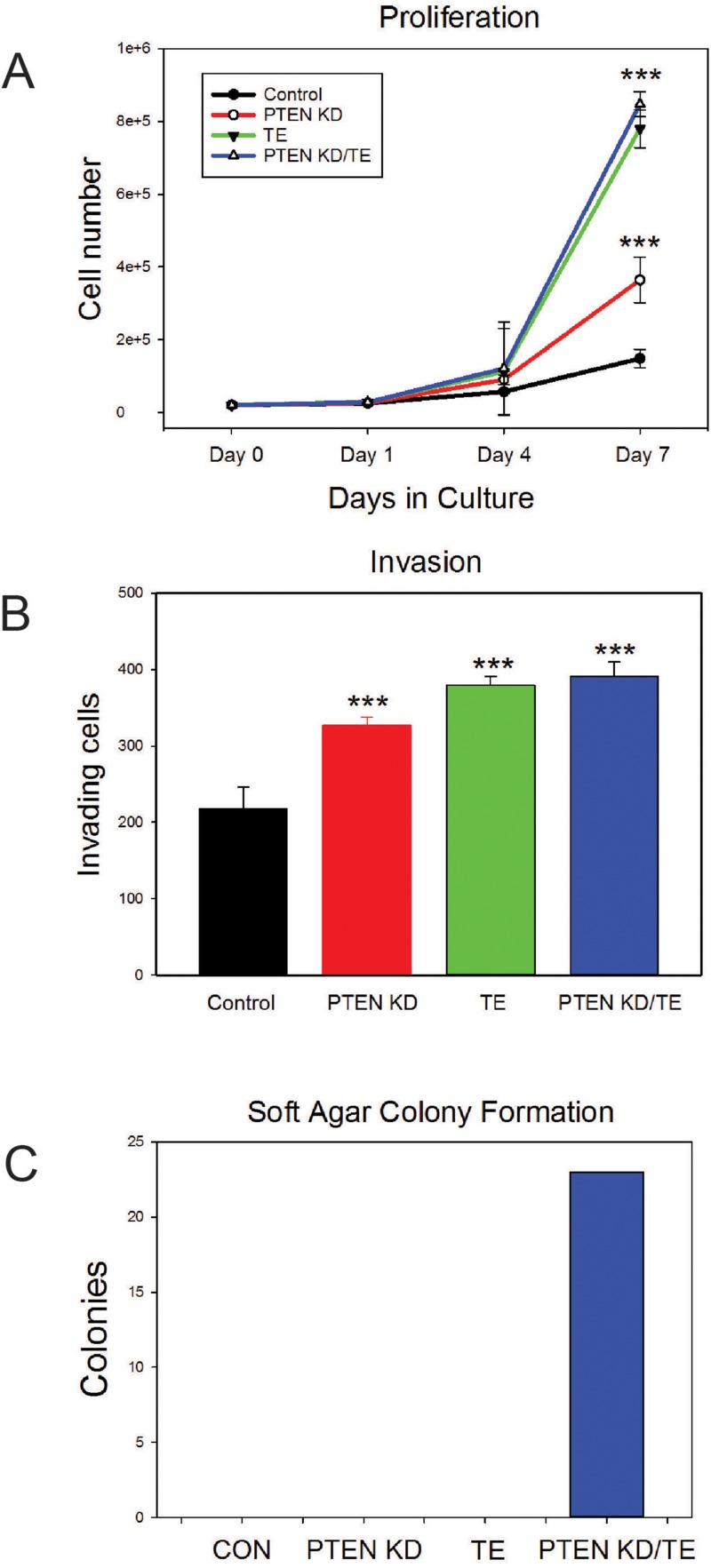
*In vitro* characterization of PNT1A cell lines with PTEN knockdown, TMPRSS2/ERG fusion gene expression or both alterations **(A)** Cell proliferation. Mean +/− SEM is shown. **(B)** Matrigel invasion. Mean +/− SEM is shown. ^***^ p<.001, t-test. (C) Soft agar colony formation.

We next evaluated the *in vitro* phenotypes of the four cell lines. The PTEN KD, TE and PTEN KD/TE cell lines all grew significantly faster *in vitro* than control cells (p<.001, t-test) although PTEN KD cells grew slower than both the TE and PTEN KD/TE cells (both p<.001, Figure 2A). Similar results were noted with invasion assays with PTEN KD, TE and PTEN KD/TE showing higher invasion than controls (p<.001) while PTEN KD showed less invasion that TE or PTEN KD/TE (p<.01; Figure 2B). Similar differences were seen in motility on plastic as assessed by a scratch assay [33] (data not shown). In contrast, only the PTEN KD/TE cells showed growth in soft agar, which is a major hallmark of the transformed phenotype *in vitro* (Figure 2C).

**Figure 3:**
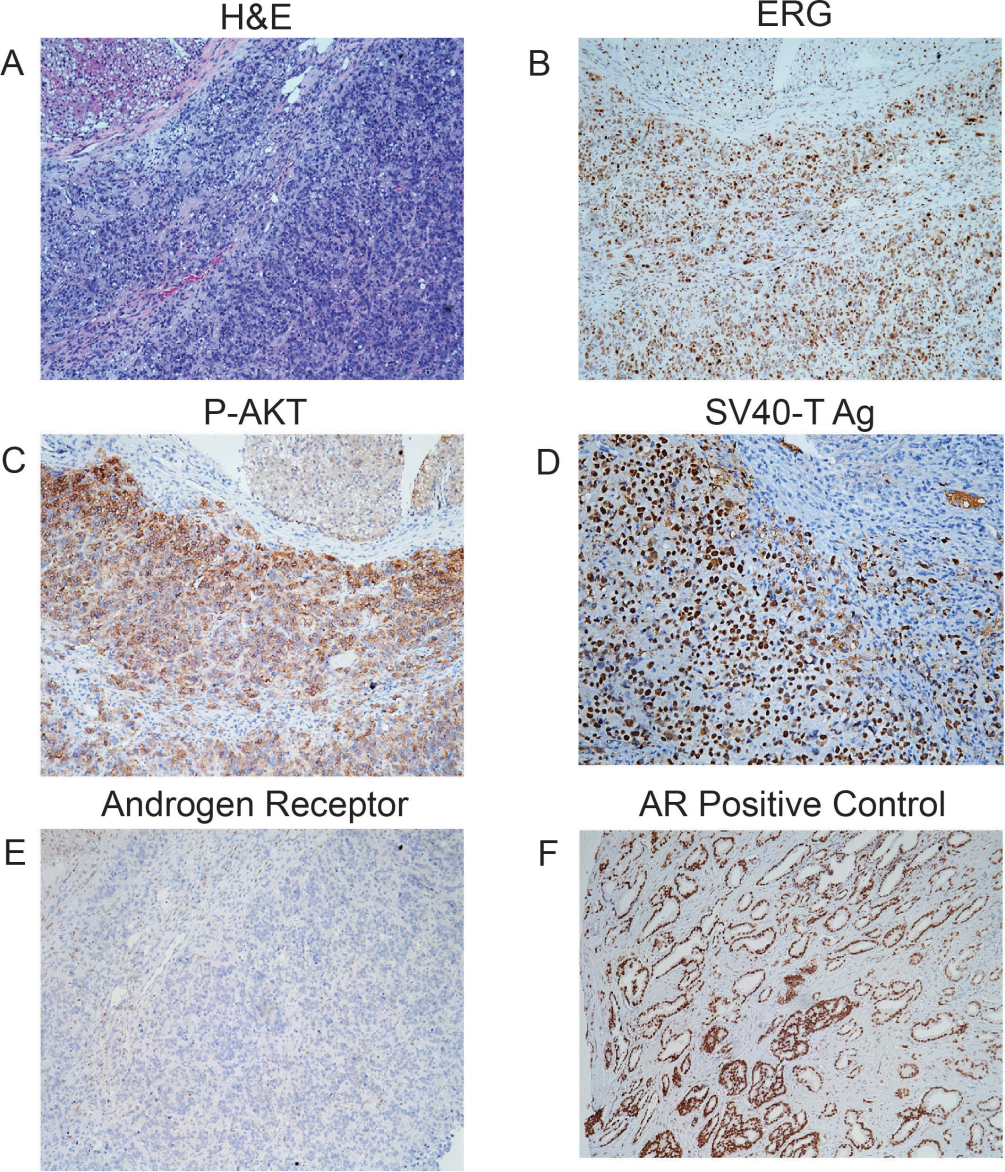
Characterization of tumors from PNT1A cells with PTEN knockdown and TE fusion gene expression Tumors from orthotopic injections of PTEN KD/TE cells were characterized by histopathology **(A)** and immunohistochemistry for ERG **(B)**, P-AKT **(C)**, SV40 T-Ag **(D)** and androgen receptor **(E)**. Positive control for androgen receptor (a human prostate cancer) is shown **(F)**.

To further evaluate the degree of transformation of the four cell lines, we carried out xenograft studies in SCID mice. In the first experiment, we injected 2×10^6^ control, PTEN KD, TE and PTEN KD/TE cells intraprostatically. After 3 months the genitourinary tracts were harvested and tumor formation evaluated by histopathology for tumor formation. Four of six mice injected with PTEN KD/TE cells had carcinomas while none of the other mice had tumors. We then carried out a similar experiment using subcutaneous injection. Three of six mice with PTEN KD/TE cells had histopathologically confirmed tumors after 3 months. The phenotype and origin of the tumors was confirmed by immunohistochemistry. All tumors expressed ERG, high levels of phospho-AKT (Ser473) and SV40-T antigen (to confirm origin from PNT1A) and were negative for AR (Figure 3). In summary, both ERG and PTEN knockdown resulted in increased growth and invasion but the combination of ERG expression and PTEN knockdown leads to the fully transformed phenotype manifested by colony formation in soft agar and tumor formation in immunocompromised mice.

### Gene expression changes associated phenotypic changes

**Figure 4:**
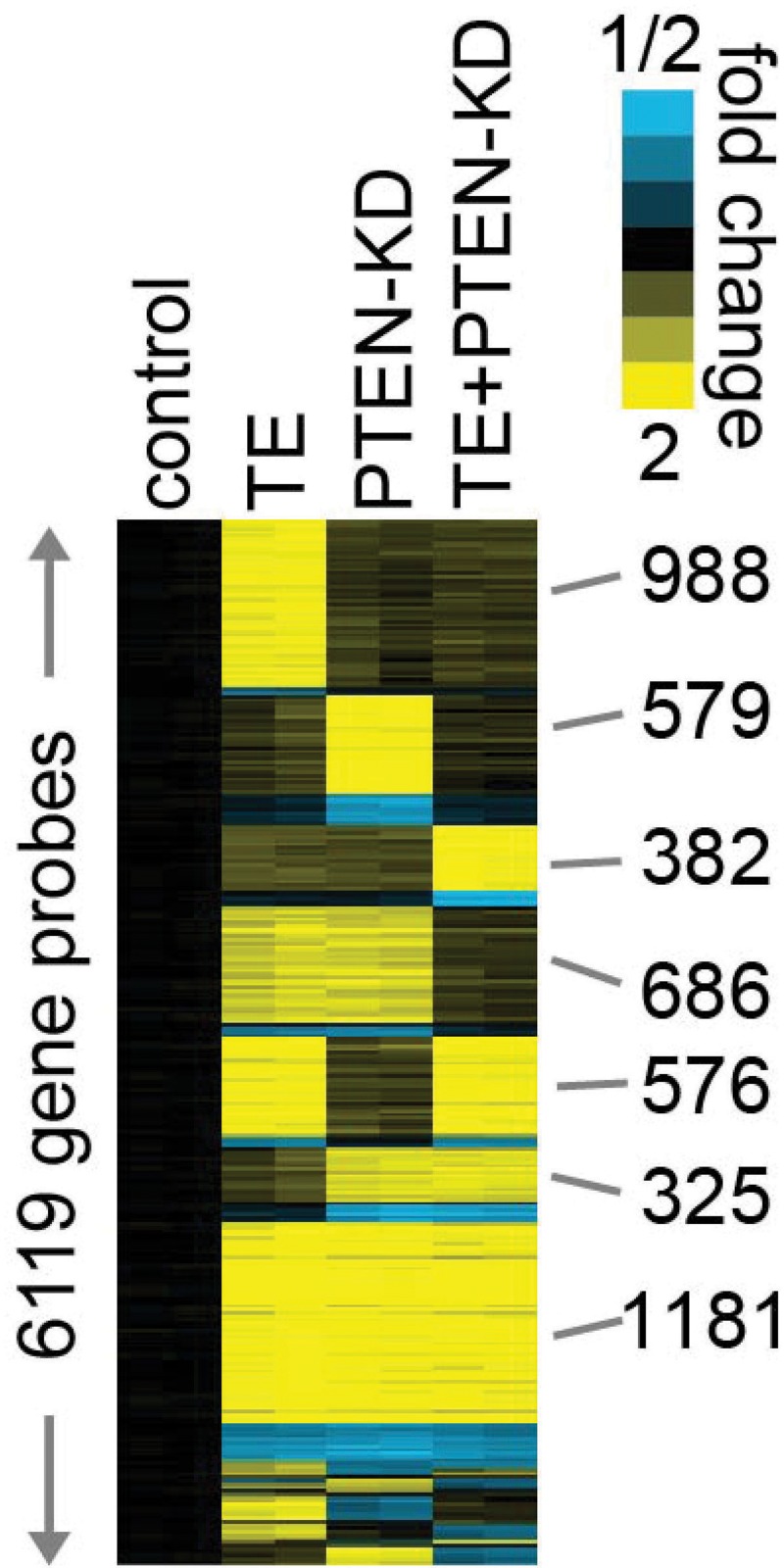
Heat map of gene expression arrays of PNT1A cell lines with PTEN knockdown and/or TMPRSS2/ERG fusion gene expression Gene expression arrays were performed on biological duplicates of PNT1A cells lines with PTEN knockdown, TMPRSS2/ERG fusion gene expression or both alterations and compared to vector controls. Probes with fold increase of >1.4 or decrease of <0.7 in at least one cell line are shown (rows, probes; columns, cell line profiles). Probes are grouped by patterns of expression in the various cell lines. Blue: decreased expression; Yellow: increased expression.

To determine what gene expression changes are associated the phenotypic changes in the four cell lines we carried expression microarray studies using Agilent 60K expression microarrays. RNAs from all four cell lines were analyzed in duplicate, and probes altered with >1.4-fold change relative to control cells (in either direction) were identified. As shown in Figure 4, a total of 6119 gene probes, corresponding to 4523 uniquely identified genes, were altered in one or more cell lines. The TE, PTEN KD and PTEN KD/TE groups each had genes that were altered (predominantly upregulated) only in that cell line. All possible combinations of altered expression pattern across the three cell lines were seen, with 181 probes upregulated in TE, PTEN KD and PTEN KD/TE groups. The PTEN KD/TE cell line had 3043 probes altered relative to controls (2471 up, 572 down), but only 382 probes were found that were unique to this cell line. A total of 343 unique protein coding (or putative protein coding) genes were altered in the PTEN KD/TE cell line, 260 upregulated and 83 downregulated (Supplementary Table 1). We reasoned that these unique changes in gene expression are critical for establishment of the fully transformed phenotype and concentrated our attention on these alterations.

### Fibroblast growth factor (FGF) and FGF receptor alterations are critical for TE and PTEN KD driven transformation

**Figure 5:**
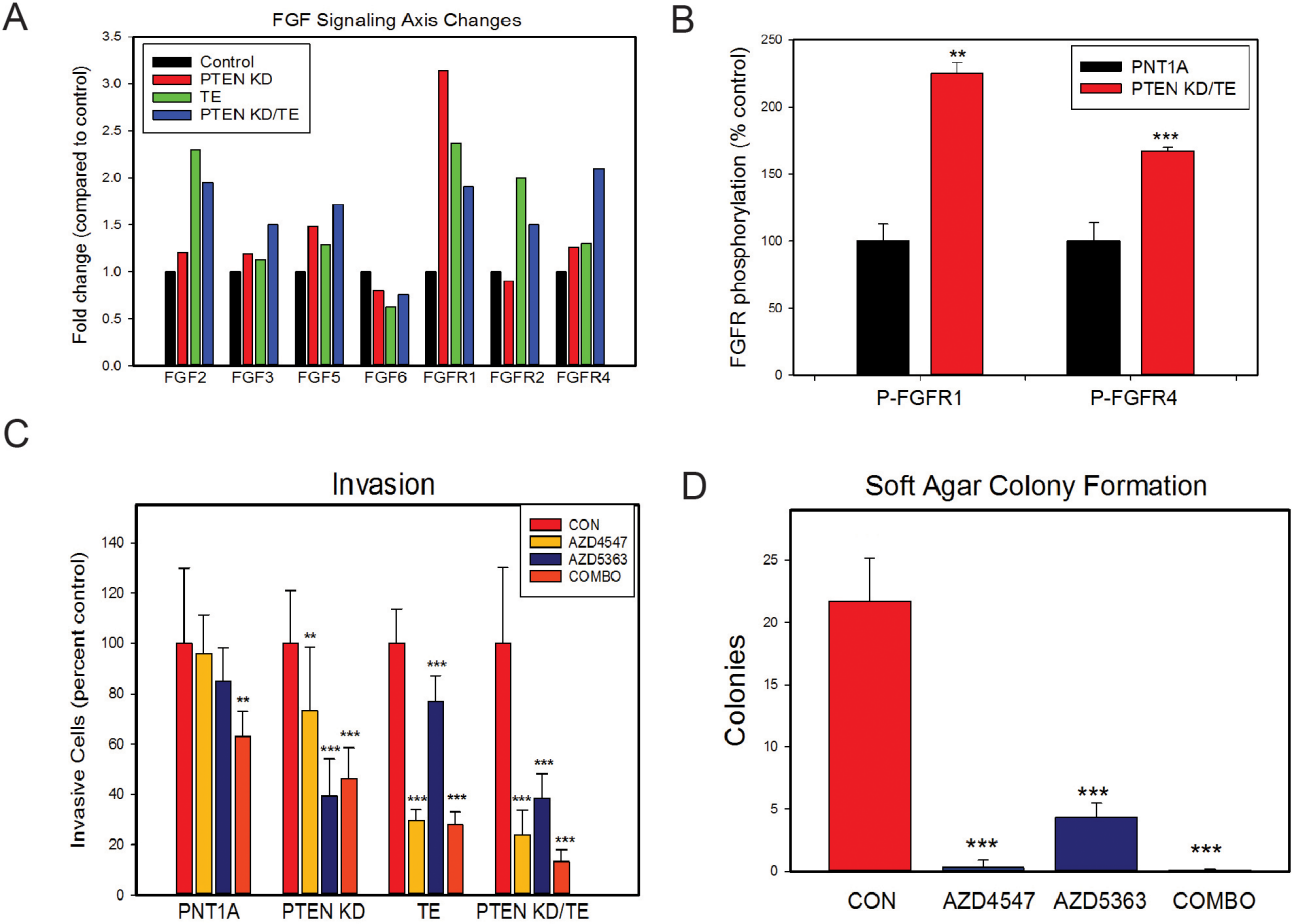
FGF signaling is increased by both PTEN knockdown and/or TE fusion gene overexpression and leads to *in vitro* changes associated with transformation **(A)** Summary of expression microarray expression of FGF ligands and FGF receptors that showed increased (1.4-fold) or decreased (0.7-fold) expression in one or more cell lines. Mean of duplicates is shown. **(B)** FGFR1 and FGFR4 phosphorylation is increased in PTEN KD/TE cells compared to control PNT1A. **(C)** Invasion through Matrigel in cell lines treated with FGFR kinase inhibitor (AZD4547), AKT kinase inhibitor (AZD5363) or both. **(D)** Soft agar colony formation of PTEN KD/TE cells treated FGFR kinase inhibitor (AZD4547), AKT kinase inhibitor (AZD5363) or both. (B-D) Means +/− SEM; ^**^ p<.01; p<.001, t-test.

Examination of the genes that were uniquely altered at the chosen thresholds in PTEN KD/TE showed that both FGF3 and FGFR4 were upregulated in these cells. Given that there is a large body of evidence that FGFs and their receptors are upregulated in PCa and that FGFR signaling is a potential drug target [26], we chose to examine the role of FGFs and FGFRs in the phenotypes observed in the TE, PTEN KD and PTEN KD/TE cell lines. FGF2, FGF3, FGF5, FGFR1, FGFR2 and FGFR4 were all upregulated at least 1.4-fold in one or more cell lines, with lower levels of increase observed as well (Figure 5A). FGF6 was the only FGF decreased 0.7-fold or more. We confirmed the increased expression of FGFR1 in the PTEN KD. TE and PTEN KD/TE cells and increased FGFR4 in PTEN KD/TE cells by Q-RT-PCR (Supplementary Figure 1A). Thus, ERG expression and PTEN KD both may increase FGFR signaling components, with potentially synergistic activation since expression of both FGF ligands and FGF receptors were increased.

We then examined both FGFR1 and FGFR4 phosphorylation the PTEN KD/TE cell lines compared to PNT1A controls after stimulation with FGF2 using phospho-FGFR ELISAs. As seen in Figure 5B, both FGFR1 and FGFR4 show significantly higher phosphorylation in the PTEN KD/TE cells. Treatment of the cell lines with an FGFR kinase inhibitor (300 nM AZD4547) markedly decreased FGFR phosphorylation, confirming the specificity of the ELISAs (Supplementary Figure 1B). Of note is that baseline FGFR1 phosphorylation in serum free media prior to FGF2 stimulation is higher in PTEN KD/TE cells than in control PNT1A (p<.01, t-test), consistent with higher autocrine FGFR1 signaling in the PTEN KD/TE cells relative to PNT1A controls. The PTEN KD cell line had higher FGFR1 phosphorylation when stimulated with FGF2 compared to control PNT1A cells but was not as high as PTEN KD/TE cells (Supplementary Figure 2A). The TE cell line had higher FGFR4 phosphorylation than PNT1A cells but again was lower the PTEN KD/TE cells (Supplementary Figure 2B). Thus, loss of PTEN and/or expression of the TE fusion gene is associated with higher basal and stimulated FGFR signaling.

To determine whether FGFR signaling plays an important role in invasion induced by TE expression and/or PTEN KD we used the FGFR kinase inhibitor AZD4547. We also used the AKT kinase inhibitor AZD5363 to confirm that the cellular phenotypes induced by PTEN knockdown are sensitive to inhibition of this key downstream target activated by PTEN loss. In control PNT1A cells there was a small but not statistically significant inhibition by either inhibitor alone but the combination inhibited invasion approximately 20% (Figure 5C). AZD4547 significantly inhibited invasion in PTEN KD, TE and PTEN KD/TE cells, confirming a role of FGFR signaling in the increased invasion cell in these cells lines. The most potent effects were seen in the TE and PTEN KD/TE cell lines. AZD5363 also inhibited invasion in all 3 cell lines and, not unexpectedly, the effects were smaller in the TE cell line. Combination treatment with both drugs was also effective in all 3 cell lines. Of note, combination treatment was no more effective than AZD4547 in TE cells and was also no more effective than AZD5363 in PTEN KD cells. However, combination treatment was more effective in the PTEN KD/TE cells than either treatment alone (Vs AZD457 alone, <.05, t-test; Vs AZD5363 alone, p<.001, t-test), with invasion being inhibited 87% by the two drugs in combination.

We then evaluated the impact of treatments single or in combination on soft agar colony formation of PTEN KD/TE cells. AZD4547 alone and in combination with AZD5363 almost completely abolished soft agar colony formation (Figure 5D). AZD5363 alone also had a profound impact on colony formation. These experiments indicate that TE expression and PTEN knockdown both increase FGFR signaling and that this signaling is critical for the transformed phenotype induced by these alterations in PNT1A cells. Of note, inhibition of FGFR signaling and/or AKT kinase activity significantly decreased proliferation in the PTEN KD/TE cells (Supplementary Figure 3) but the effects were smaller than those seen on soft agar colony formation. Thus, the impact of inhibition of these kinase pathways on soft agar colony formation was only partially through inhibition of proliferation.

**Figure 6:**
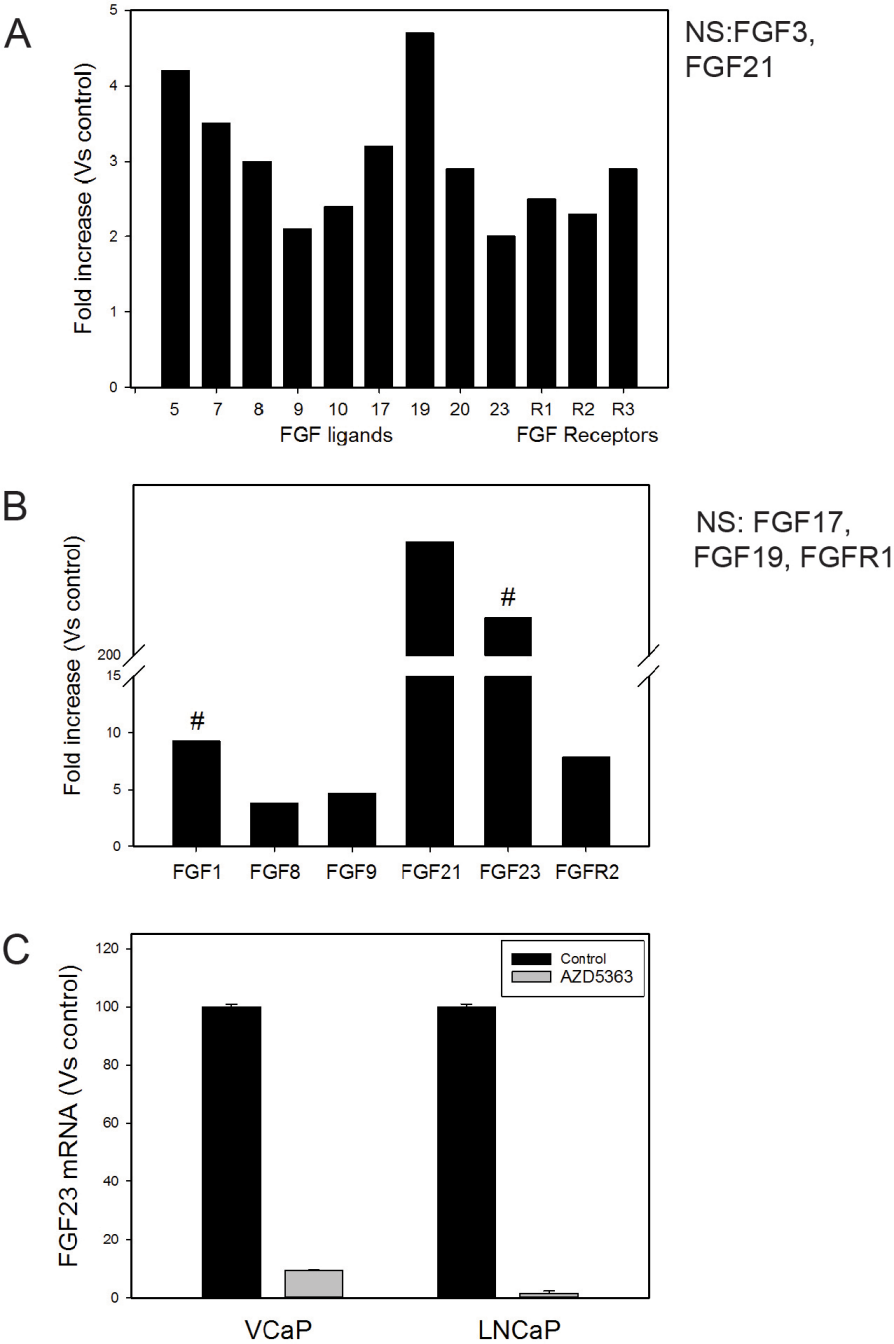
Decreased PTEN increases expression of FGF ligands and FGF receptors **(A)** Fold increase of FGF ligands and FGF receptors in 22RV1 cells with PTEN knockdown versus controls. **(B)** Fold increase of FGF ligands and FGF receptors in VCaP cells with PTEN knockdown versus controls. For FGF1 and FGF23 fold-increase was calculated assuming a Ct value of 40 cycles since both ligands were not detectable in the assay; this is indicated by #. For both 22RV1 and VCaP cells FGF ligands or FGF receptors that were altered but not 2-fold or 0.5-fold are shown as NS (not significant). **(C)** FGF23 mRNA levels after treatment of VCaP or LNCaP with the AKT kinase inhibitor AZD5363. Mean +/− SEM is shown.

We then examined whether PTEN knockdown altered FGF signaling in PCa cell lines. We analyzed RNAs from 22RV1 cells (which have wild-type PTEN) and 22RV1 with PTEN knockdown using a human FGF pathways Q-RT-PCR array (Thermo Fisher) to identify changes in FGF ligands and/or receptors induced by PTEN knockdown, with increases of 2-fold or decreases 0.5-fold considered biologically significant. As shown in Figure 6A, PTEN knockdown resulted in increased ligands and receptors from 2.0 to 4.7-fold including FGF5, FGF7, FGF8, FGF9, FGF10, FGF17, FGF20, FGF23 and FGFR1, FGFR2 and FGFR3 (Figure 6A). No FGFs or FGFRs were significantly decreased. FGF11-14 are not shown as they are not FGF ligands. It should be noted that FGF6, FGF15 (mouse homolog of FGF19), FGF16, FGF18, FGF22 and FGFR4 are not represented on the array. We carried out a similar experiment using VCaP cells, which are PTEN wild type, with PTEN knockdown (80% knockdown by Q-RT-PCR). As shown in Figure 6B, multiple FGF ligands and receptors were upregulated 3.8 to 230-fold relative to control cells including FGF1, FGF8, FGF9, FGF21 and FGF23 and FGFR2 was increased 7.9-fold. No FGFs or FGFRs were decreased. We confirmed the marked increase of FGF23 in the VCaP PTEN knockdown cells (versus controls) using Q-RT-PCR (not shown). We then treated VCaP cells with the AKT kinase inhibitor AZD5363 and observed a marked decrease in FGF23 mRNA (Figure 6C). In addition, we observed a marked decrease in FGF23 mRNA when LNCaP (mutant PTEN) were treated AZD5363 (Figure 6C). Overall this data supports the concept that PTEN knockdown increases FGF and FGFR expression in a pleiotropic manner by altering AKT activity.

**Figure 7:**
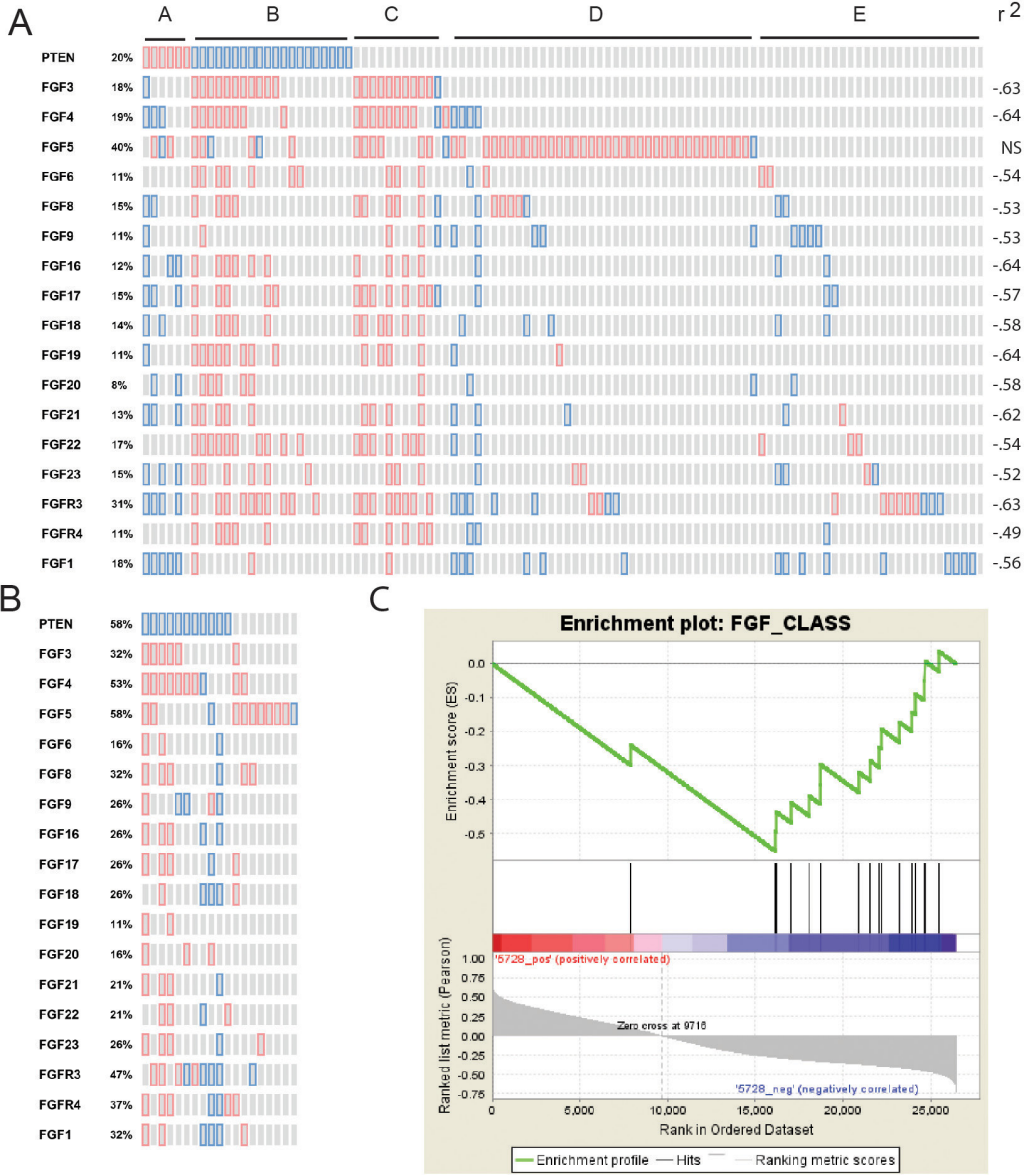
Correlation of loss of PTEN expression with increased FGFs and FGF receptors **(A** and **B)** Expression of PTEN, FGF ligands and FGFR3 and FGFR4 was analyzed in cBioportal. Genes are indicated for each row. Red shows increased expression and blue loss based on z-scores versus normal of 1.4 or greater. (A) Primary tumors with mRNA are shown in the columns Spearman correlation of mRNA expression of each FGF and FGFR with PTEN is shown on the right. (B) Metastatic tumors with mRNA are shown in columns. **(C)** Gene Set Enrichment Analysis of FGF ligands and FGFR3 and FGFR4 in PCas from the Taylor dataset showing association of expression of FGF signaling components with loss of PTEN. Genes in the Taylor dataset are ranked from high to low Pearson's correlation with *PTEN* (Entrez ID 5728). Positions of the FGF ligands and *FGFR3* and *FGFR4* are indicated along this ranked list.

To determine whether there is evidence of an association of PTEN loss with expression of FGF signaling components in human PCa specimens we examined mRNA expression of human FGF ligands and FGF receptors in 131 primary cancers with mRNA with from the Taylor dataset [34] in cBioportal [35]. We excluded FGF2, FGF7, FGF10 and FGFR1 from this analysis since they are expressed in benign stroma and FGFR2 is expressed in benign epithelium. This expression makes analysis of these mRNAs in cancer cells difficult since variable admixture of benign stroma and epithelium obscures any correlations. This is confirmed by the finding that these RNAs are negatively correlated with alpha-methylacyl-coA racemase (AMACR), the best mRNA marker for PCa, and positively correlated with FGF7, a known stromal marker [36] (Supplementary Table 2). As shown in Figure 7A, all FGF ligands (except FGF5), FGFR3 and FGFR4 are all strongly negatively correlated with PTEN expression (−.49 to -.64, Spearman). The primary PCa cases fall into 5 groups as shown in Figure 7A. Group A shows increased PTEN and this is associated with lower expression of multiple FGF ligands (except FGF5) and FGFR3. Group B shows decreased PTEN and increased expression of multiple FGF ligands, with up to 13 ligands being overexpressed, and overexpression of FGFR3 and FGFR4. Group C shows a similar pattern of gain of FGF ligands and receptors but no loss of PTEN. We hypothesize that these cases may have alternative modes of activation of the PI3K/AKT pathway. Group D is characterized by numerous cases with upregulation of FGF5. Group E shows only scattered gains and losses of FGF ligands and FGFR3. Thus, loss of PTEN is strongly linked to upregulation of multiple FGF ligands (except FGF5) as well as FGFR3 and FGFR4. FGF5, which is increased in a significant fraction of cases, is not correlated with PTEN loss, although it is increased in some cases with PTEN loss.

Examination of the smaller number of metastatic cases with mRNA revealed a similar pattern. As seen in Figure 7B, 16 of 19 metastatic cases had upregulation of one or more FGF ligands, with up to 13 ligands increased in some cases, similar to our previously reported finding in the metastatic cancers analyzed at Fred Hutchison Cancer Center [26]. PTEN loss was much more common in the metastatic cases, as is well known [34]. Statistical power for analysis of individual FGF ligands was limited due to the small number of cases so we carried out a chi-squared analysis of increased FGF ligands and FGFRs in these cases. Examination of the 11 cases with PTEN loss showed overexpression of an FGF ligand (excluding FGF5) in 42 instances of a maximum possible of 154 (11 cases X 14 ligands). Of the 8 cases with retention of PTEN, there were 9 instances of increased expression of FGF ligands (other than FGF5) of 88 possible instances. This difference was highly statistically significant (p<.01, chi-squared). On the other hand, 7 of 8 cases without PTEN loss showed increased FGF5, while only 2 of 11 with PTEN loss showed increased FGF5 (p <.01, Fisher exact test). Thus, in metastatic cases there is widespread increases in expression of FGF ligands with cases having decreased PTEN expression showing increased FGF ligands other than FGF5 and cases with retention of PTEN commonly expressing FGF5.

We also carried out Gene Set Enrichment Analysis using the FGF ligands and FGFRs described above on the Taylor dataset of primary and metastatic PCa. We found a highly significant enrichment of FGF ligands and FGFRs in cases with PTEN loss (p<.001; Figure 7C). All the individual FGF ligands (except FGF5) and the FGFRs were negatively correlated with PTEN. Thus, our in silico analysis shows that PTEN loss is associated with increases in multiple FGF ligands and FGFRs, consistent with our analysis is prostate and PCa cell lines.

## DISCUSSION

Understanding the basis of the ability of PTEN loss to promote transformation by TE fusion gene is critically important since these two alterations are the most common genomic alterations observed in PCa and are often associated with each other in human PCa. We demonstrate here that PTEN loss complements TE fusion gene expression to fully transform immortalized prostate epithelial cells. These cells do not express AR and thus, at least in the context of immortalized cells, AR is not required for transformation induced by PTEN loss and ERG overexpression. However, as shown by Chen et al [20] it is clear that ERG has significant impact on AR transcription in the face of PTEN loss and that these transcriptional changes almost certainly have wide ranging impacts on PCa biology. Our studies indicate that PTEN loss and TE expression can lead to widespread changes in gene expression and activate pathways altered in PCa progression independent of AR signaling and can result in full transformation. Among these pathways, FGFR signaling is critical, since inhibition of FGF signaling has a profound impact on the transformed phenotype in this context.

It should be noted that in in genetically engineered mouse models a TMPRSS2/ERG transgene containing the TMPRSS2/ERG promoter was expressed in basal cells, which have weak, if any, AR signaling [37]. Such cells have been shown to give rise to PCa in the PTEN knockout model [38]. Basal cells were also capable of PCa initiation using *in vivo* prostate regeneration assays [39]. Thus, analysis of transformation by the TE fusion gene expression in the absence of AR activity is relevant to prostate carcinogenesis *in vivo*.

Our *in vitro* and in silico data show that loss of PTEN, presumably by activation of AKT, can enhance expression of multiple FGF ligands and FGF receptors. This increased expression is highly variable, with different ligands and receptors increased in different cell lines or tumors. This indicates that the impact of PTEN loss on expression of each ligand and receptor is likely to be highly context dependent. Previous studies by our group and others [21, 22, 25–27, 29, 31] individual ligands and receptors have shown multiple ligands and receptors are increased in PCa. Our recent collaborative bioinformatic study of metastatic prostate cancers based on RNA sequencing showed a pattern like our current study; almost ubiquitous but highly variable expression of FGF ligands and receptors in these cancers [26]. Given that PTEN is lost or AKT is activated by alternative mechanisms in the vast majority of advanced PCas [34], our current studies provide a reasonable explanation for these observations. It should be noted that the FGF2 and FGFR1, the two main targets of the TE fusion are not amenable to our in silico analysis due to their high expression in stroma, However, we did not find evidence of induction of other FGFs by TE fusion gene using our in silico approach.

Activation of FGF signaling by loss of PTEN has been reported in bone. Guntur et al [40] has shown that PTEN deletion in osteoprogenitors results in increased FGF18 and increased osteoblasts. The phenotype could be partially rescued by deletion of one allele of FGFR2. Deletion of the phosphatase Phlipp1, which targets components of the AKT pathway, also increased FGF18 and had a similar phenotype in osteoprogenitors [41]. FGF18 induction was due to decreased FOXO1 activity. Whether FOXO1 is involved in repression of FGF18 or other FGF signaling components in PCa is not known. Clearly, additional studies are needed to understand how PTEN loss increase FGF signaling in PCa.

Our studies show increased expression of multiple FGF ligands at the mRNA level after PTEN loss. Two studies have shown increased translational efficiency of FGF ligands including FGF2 [42] and FGF10 [43] after PTEN loss. Such changes in translational efficiency have the potential to further increase FGF ligand availability after loss of PTEN. Additional studies are needed to determine the importance of such increases in translational efficiency on transformed phenotypes induced by PTEN loss in PCa.

It is of interest to note that FGF5 was the one exception to our finding that PTEN loss is associated with FGF ligand expression. There is little literature on the expression of FGF5 in PCa [44]. However, data from our current study our prior in silico analysis [26] indicates that it is expressed commonly in both primary and metastatic PCa. It may represent an alternative pathway to activate FGF signaling in those PCas without PTEN loss and/or AKT activation, although at least in some contexts (such as 22RV1 cells), it can be induced by decreased PTEN activity. FGF5 can also be induced by hedgehog signaling [45] and SOX2 [46], so perhaps these may represent an alternative pathway for induction FGF5 in PCa.

Our *in vitro* data indicates that inhibition of FGF signaling has a profound impact on transformation related phenotypes in the TE and PTEN KD/TE cell lines. Invasion was decreased ~80% in both cell lines by treatment with AZD4547. More importantly, soft agar colony formation was almost eliminated in the PTEN/KD TE cell. However, PTEN KD cells had smaller decreases in these phenotypes in response to FGFR inhibition, despite the increase in FGFs and FGF ligands seen. The reason for this is unclear. FGF2 was particularly increased by TE expression and this FGF is a known contributor to the transformed phenotype in PCa [22]. As noted above, FGF2 translation is increased after PTEN knockdown [42] so this may lead to synergistic effects of PTEN KD and TE expression. This is accompanied by increased FGFR1, which binds FGF2, and is also strongly associated with transformation in PCa [22, 24]. FGFR4 was induced synergistically by PTEN loss and TE fusion gene expression and our prior studies have strongly implicated FGFR4 in PCa progression [23, 28, 47]. It also possible that other changes in gene expression, such is increased FGF binding proteins (which enhance FGF signaling) or decreased negative regulators of FGFR signaling may be induced by TE fusion gene expression and result in higher FGFR signaling. Alternatively, pathways that synergize with FGFR signaling in producing the observed phenotypes may be induced by the TE fusion gene. Additional studies are needed to clarify the reasons for the observed phenotypes and responses to FGFR inhibition.

In summary, our study shows that FGFR signaling plays a very important role in transformation induced by loss of the PTEN tumor suppressor, particularly when combined with expression of the TE fusion gene. Given that multiple, relatively low toxicity FGFR receptor inhibitors are entering the clinic our data suggests that they may be useful for treating PCa with the TE fusion and PTEN loss. Reliable immunohistochemical protocols are available to identify men with these two alterations [48] allowing straightforward patient selection. Of course, other pathways are also activated in this context, so additional therapies will probably be needed as well to obtain optimal outcomes.

## MATERIALS AND METHODS

### Cell culture

PNT1A and 22RV1 cells were maintained in the RPMI with 10% fetal bovine serum (FBS). VCaP and HEK 293T cells were cultured in DMEM with 10% FBS. PNT1A cells were obtained from the European Type Culture Collection. LNCaP, VCaP and 293T cells were obtained from the American Type Culture Collection. Cells were obtained between 2001 and 2016, expanded, frozen and stored as stocks in liquid nitrogen. All cell lines are authenticated by STR analysis at MD Anderson Cancer Center Characterized Cell Line Core Facility.

### Generation of stable PNT1A cell lines

GIPZ shRNA clones targeting human PTEN gene and non-silencing pGIPZ control vector, both containing the Turbo GFP reporter, puromycin-resistance gene, and elements required to allow packaging of the expression construct into virions were purchased from the Cell-Based Assay Screening Core Facility at Baylor College of Medicine. The mature antisense sequences are: *5’-*AATGTTTGGATAAATATAG-3’ (Clone Id:V2LHS_92314) and 5’-TAATAATACACATAGCGCC-3’ (V2LHS_92317). Lentiviral shRNA was produced by cotransfection of the Trans-Lentiviral packaging mix with a shRNA transfer vector into 293T packaging cells.

The TE isoform (3+72) lentivirus [33] was constructed as follows. Exons 1-3 of TMPRSS2 fused to ERG exon 4) fragment containing TMPRSS2 sequence 12 to 71 nt (NM 005656) and ERG sequence 226 to 762 (NM 004449) was subcloned into pcDH-CMV-EF1-neomycin vector (CD510B-1, System Biosciences). 20 ng of the plasmid DNA 3.1/V5-His-TMP/ERG 3+72 [33] was used as template for PCR using primer pair: F NheI: 5’- CCCT CGTATCGCTAGCGCGAGCTAAGCAGGAGG −3’ and R NotI: 5’-CCGTAGATCG GCGGCCGC TTAGTAGTAAGTGCCCAGATGAG-3’ at 60 °C. The Q5® Hot Start High-Fidelity 2X Master Mix (NE Biolabs) was used for the PCR reaction. The absence of mutations was confirmed by sequencing. Amplified fragments were digested by NheI and *Not*I and ligated into pcDH-CMV-EF1-neomycin vector. TE lentiviral particles were generated in 293T cells using pCDH-CMV-MCSEF1- TE3+72-neo and pPACK Lentivector packaging kit (System Biosciences).

To make stable cell lines, RPMI medium supplemented with lentiviral supernatants PTEN KD, TE or both were supplemented with 5 μg/mL polybrene and incubated with cells for 24 hours. The PTEN KD cells were maintained in medium with 400ug/mL puromycin. PNT1A stably transfected with PTEN shRNA with clone Id V2LHS_92314 and V2LHS_92317 were used for *in vitro* experiments. V2LHS_92317 were used for the *in vivo* studies because of their better PTEN knockdown efficiency. Similarly, PNT1A with TE expression were generated by infecting with TE lentivirus and selected in 200ug/mL G418. The combined PTEN KD/TE stable cell line was generated by infection with equal amounts of TE and PTEN shRNA lentivirus and selected with puromycin and G418. The cell lines were generated twice and had the same *in vitro* phenotypes both times.

### Evaluation of FGF and FGFR expression in PCa cell lines

To evaluate the role of PTEN in controlling FGF and FGFR receptor expression a VCAP cell with stable PTEN knockdown was constructed as described for the PNT1A stable cell lines using PTEN ShRNA lentivirus (V2LHS_92317) and vector control. Q-RT-PCR showed a 80% knockdown efficiency. For 22RV1 cells PTEN was knocked down transiently using PTEN siRNA purchased from Sigma; PTEN sense: 5’-AAC CCA CCA CAG CUA GAA CUU dTdT-3’ and antisense: 5’-AAG UUC UAG CUG UGG UGG GUU dTdT-3 and scramble siRNA control. Transfections were performed using 50 nM with RNAiMax transfection reagent (Invitrogen) for 48 hours and RNA was extracted. PTEN mRNA knockdown was 75% compared to controls. To evaluate FGF23 expression LNCaP or VCaP, cells were treated for 48 hours with 300 nM AZD5363 or vehicle, RNA was extracted and used for Q-RT-PCR of FGF23 mRNA.

### cDNA synthesis and quantitative real-time PCR (Q-RT-PCR)

Total RNA was isolated using the RNeasy Mini Kit (Qiagen). 500 ng total RNA were used for cDNA reverse transcription using amfi Rivert Platinum cDNA Synthesis Enzyme Mix (GenDEPOT). 1-5 uL of cDNA was used for Q-RT-CR using in a final reaction volume of 15ul using SYBR Green PCR Master Mix (Applied Biosystems) for PTEN or TaqMan Fast Advanced Master Mix (Applied Biosystems). The PTEN primers were sense: 5’- AGCGTGCAGATA ATGACAAGG-3’ and antisense: 5’- TGGATCAGA GTCAGTGGTGTC-3’, with an annealing temperature of 60° C. FGFR1 and FGFR4 were analyzed using Taqman MGB probe (FAM-FGFR1 and FGFR4 and VIC-ACT). FGF23 primers and conditions were used as published in previously [25]. Q-RT-PCR was carried out in a StepOnePlus™ real-time thermal cycler (Applied Biosystems) using standard parameters. Each experiment was performed in triplicate and the differences in expression levels were evaluated using 2- ΔΔCT method. Expression data were normalized to β-actin.

### Western blotting

Cells were lysed in modified RIPA buffer (Santa Cruz) supplemented with PMSF, protease inhibitor and sodium orthovanadate. Protein concentration of the lysates were determined using BCA protein assay reagent (Bio-Rad); 30μg of the extracted protein was mixed with Laemmli sample buffer containing β-ME, denatured, separated using 10% PAGEr Gold Precast Gels (Lonza), and transferred using iBlot Gel Transfer system (Invitrogen) and iBlot Gel Transfer Stacks Nitrocellulose (Invitrogen). Anti-ERG (#2805-1, Epitomics), anti-PTEN (#S-0271, Epitomics), anti-β-actin antibody (Santa Cruz Biotech), antibodies were used at 1:1000 dilution with blocking with 5% skim milk. After incubation with primary antibodies overnight at 4°C, horseradish peroxidase–labeled secondary antibodies were then applied to the membranes for 1 h at room temperature. Signals were visualized using ECL Western blotting detection substrate (Thermo Scientific).

### Proliferation assays

1×10^5^ cells were seeded on 24-well plates in triplicate and attached cells were counted using Beckman Cell counter. The experiment was repeated three times. Final experiments were also confirmed by MTT assay using CellTiter 96 Aqueous One Solution Cell Proliferation Assay (Promega).

### Matrigel invasion assays

Invasion assays were conducted using pre-coated BD Matrigel Invasion Chamber 24 well plates (BD Biosciences) in triplicate. A total of 5000 cells were plated per insert and after 48 hrs culture, non-invading cells in the upper chambers were removed and the invading cells on the lower surface of the membrane were fixed and stained with DAPI. The membranes were mounted on slides, photographed under fluorescent microscopy at 10X and cells enumerated using Image J software. Each experiment was repeated 3 times. For drug treatment AZD4547 or AZD5363 was added in media in the bottom wells with the final concentration of 300 nM for both drugs as described previously [26].

### Soft agar colony formation assays

Five thousand cells expressing were mixed with the 0.7% agarose (top agar) with warm 2×RPMI 1640 + 20% fetal bovine serum and plated in each well of a 6-well plate on top of the prepared 1% base agar. Plates were incubated at 37°C for 3 weeks before the foci were stained with 0.005% Crystal Violet and counted. For drug treatment, AZD4547 or AZD5363 was supplemented to the 2XRPMI media for the preparation of the base agar and top agar with the final concentration of 300 nM. Twice a week 100 uL of RPMI medium supplemented with 300 nM of one or both drugs was added to the top of the agar to keep the agar humid.

### Immunohistochemistry

Immunohistochemistry (IHC) was carried out using the general procedures described previously [49], using steam with Tris-EDTA buffer (ph 9.0) for antigen retrieval [14]. Detection was carried out at room temperature using a PolyVue Polymer detection system (Diagnostic Biosystems) according to manufacturer's directions. Primary antibodies were: anti-ERG antibody (#2805-1, Epitomics); phospho-AKT S473(#9271, Cell Signaling); androgen receptor (clone EPR 1535(2), Cat#3165-1, Epitomics); SV40 T Ag (Santa Cruz v-300, sc-20800); all at 1:100 dilution for 30 min at room temperature.

### Phospho-FGFR ELISAs

Phospho-FGFR1 levels from the cells were measured using PathScan® Phospho-FGF Receptor 1 (panTyr) Sandwich ELISA Kit (#12909, Cell Signaling) according to manufacturer's protocol. The cell lysates were prepared using cell lysis buffer included in the kit supplemented with 1mM PMSF, sonicated, spun down at 4 °C and supernatant protein concentration was measured using BCA method (Bio-Rad.). A total of 300 ug protein was loaded to the ELISA strip in duplicate in total volume of 200 uL adjusted with sample buffer included in the kit. After incubations and washes following the manufacturers protocol optical density at 450 nM was measured using a microplate reader (VERSAmax tunable, Molecular Devices). The positive control was 30 ug of A201 treated with FGF (provided by Cell Signaling) and 200 ug of 22RV1 cells lysate treated with 300 nM AZD4547 was served as negative control. Similarly, Phospho-FGFR4 (PanTyr) ELISA kit (#69193, Cell Signaling) was used for Phospho-FGFR4 detection in cell lysates.

### Xenograft studies

All procedures were approved by the Baylor College of Medicine Institutional Animal Use and Care Committee. In the first experiment, eight-week old male SCID mice were injected intraprostatically with 2×10^6^ PNT1A TE, PNT1A-PTEN KD, PNT1A PTEN KD/TE in or PNT1A vector control cells in SCID mice and after 3 months the genitourinary tracts of all mice were harvested and H&E sections evaluated for tumor formation by a pathologist (MI). In a 2nd experiment, SCID mice were injected subcutaneously over both lateral flanks with 3×10^6^ cells of each genotype in 200ul volume mixed with 100ul Matrigel (BD Bioscience). After 3 months, any palpable tumors were collected fixed and paraffin-embedded for H&E examination to confirm the presence of cancer cells.

### Microarray gene expression analysis

Total RNA were extracted with RNeasy Mini Kit (Qiagen), according to the manufacturer's recommendations. The cDNA reverse transcription and fluorescent labeling reactions were carried out using Invitrogen SuperScript Plus Direct cDNA Labeling System with Alexa Fluor S’-Aminohexylacrylamido-dUTP. 60K Whole Human Genome Oligo Microarray chip (Agilent Technologies) using SureHyb DNA Microarray Hybridization Chambers as described previously [25].

For each treatment group, top differentially expressed genes relative to control were defined (using fold change >1.4 for each experimental profile compared to each control profile), and the set of top differential genes found for any treatment group were clustered, using a supervised approach as described elsewhere [50]. Expression patterns were visualized as color maps using Java TreeView [51]. Array data have been deposited into the Gene Expression Omnibus (GSE101635). Expression arrays were processed using Bioconductor (with loess normalization). Gene Set Enrichment Analysis (GSEA) was carried out [52] using Pearson's correlation as the ranking metric, and with the “classic” scoring scheme.

### FGF pathway Q-RT-PCR arrays

TaqMan™ Array Human FGF Pathway (ThermoFisher, cat#4414136), which contains 92 assays to FGF pathway associated genes and 4 assays to candidate endogenous control genes, was used to evaluate FGF ligands and/or receptor changes in 22RV1 and 22RV1 with PTEN knockdown or VCaP and VCaP with PTEN knockdown cell lines. 25ng of cDNA was added into each well of these 96 wells FGF arrays along with TaqMan® Gene Expression Master Mix (2x) to make the total reaction of 10ul. Real-time PCR were performed using Thermo Fisher StepOnePlus™ by standard protocol.

### Statistical analysis

Numerical values were compared using t-test. Proportions were compared with chi-squared test or Fishers exact test. Differences were considered significant if p<.05.

Bioinformatics analysis: Yiqun Zhang and Chad J. Creighton.

Study supervision and data analysis: Michael Ittmann.

## SUPPLEMENTARY MATERIALS FIGURES AND TABLES





## Abbreviations

PCa: prostate cancer
TE: TMPRSS2/ERG
FGF: fibroblast growth factors
KD: knockdown
