# Supplementary material for: Fibroblast growth factor receptor signaling plays a key role in transformation induced by the TMPRSS2/ERG fusion gene and decreased PTEN
Source: Oncotarget. 2018 Feb 12;9(18):14456–71. doi: 10.18632/oncotarget.24470 (PMC5865682; doi:10.18632/oncotarget.24470)
Supplement: Supplementary file 1 [file oncotarget-09-14456-s001.pdf]

Fibroblast growth factor receptor signaling plays a key role in transformation induced by the TMPRSS2/ERG fusion gene and decreased PTEN

SUPPLEMENTARY MATERIALS

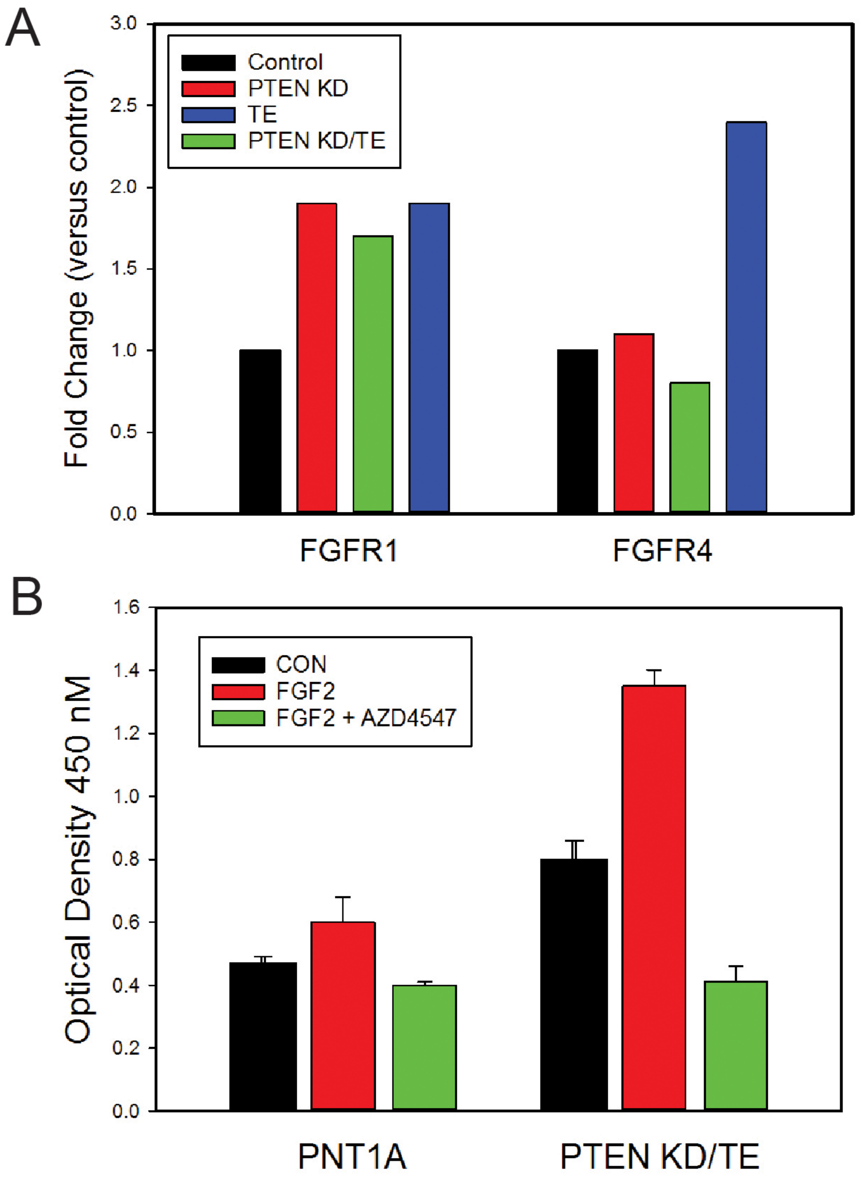

Supplementary Figure 1: Increased expression and activity of FGFRs due to decreased PTEN and/or expression of the TMPRSS2/ERG fusion gene. **(A)** Expression of FGFR1 and FGFR4 mRNA in PNT1A, PTEN KD, TE and PTEN KD/TE cells. Expressed as fold change relative to PNT1A control cells. Means of duplicate or triplicate determinations by Q-RT-PCR is shown. **(B)** Optical density reading of phospho-FGFR1 ELISA of PNT1A or PTEN KD/TE cells treated with vehicle, FGF2 or FGF2 in the presence of FGF kinase inhibitor AZD4547. Means +/- standard deviation of triplicate determinations are shown.

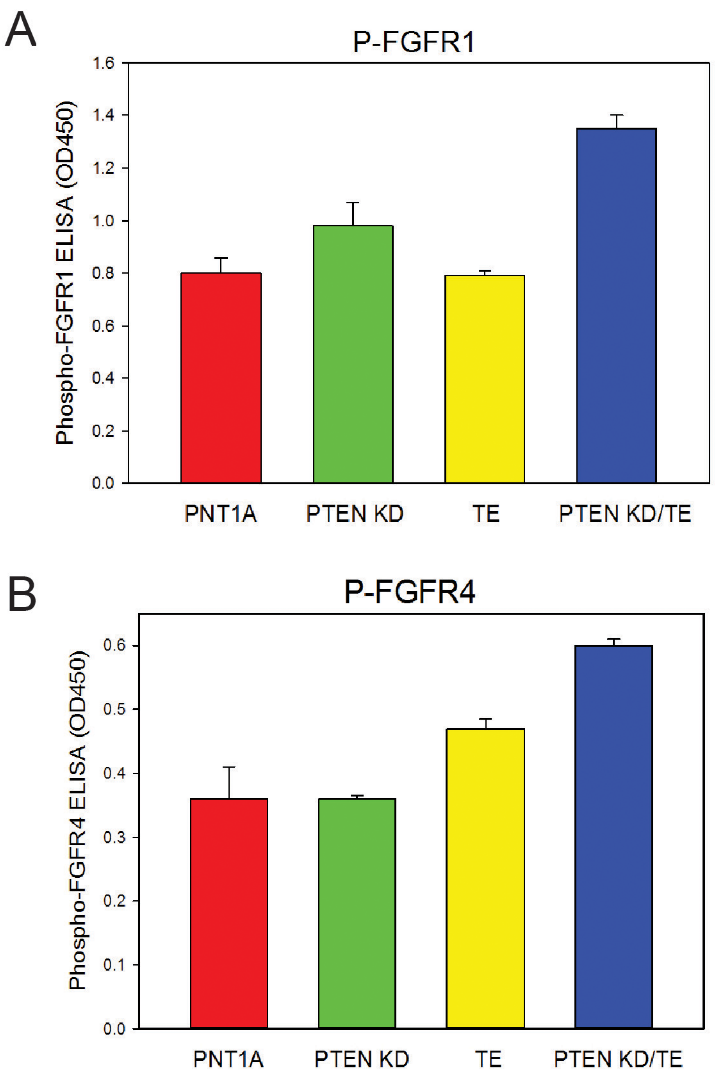

Supplementary Figure 2: Increased FGFR phosphorylation in PTEN KD, TE and PTEN KD/TE cells. **(A)** Optical density reading of phospho-FGFR1 ELISA for PNT1A, PTEN KD, TE and PTEN KD/TE cells treated with FGF2 **(B)** Optical density reading of phospho-FGFR4 ELISA for PNT1A, PTEN KD, TE and PTEN KD/TE cells treated with FGF2. Means +/- standard deviation of triplicate determinations are shown.

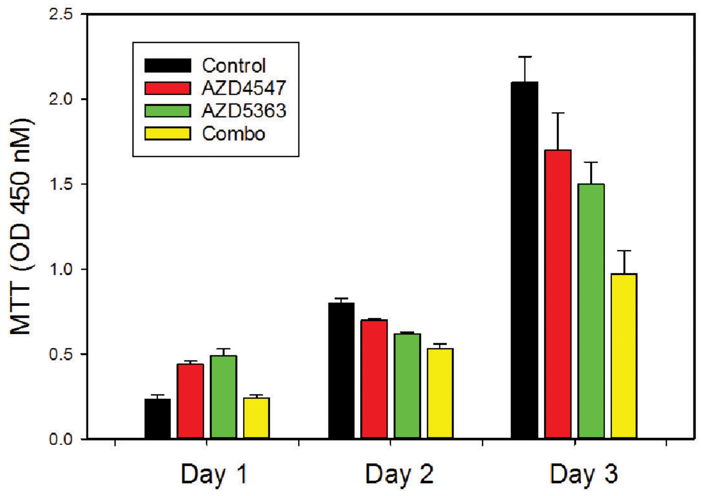

Supplementary Figure 3: Proliferation of PTEN KD/TE cells treated with AZD4547, AZD5363 or both inhibitors. Optical density of MTT assays at days 1-3 after initiation of treatment are shown. All treatments at days 2 and 3 are statistically significant versus vehicle by t-test. Means +/- standard deviation of triplicate determinations are shown.

Supplementary Table 1: Protein coding genes altered in only the PTEN KD/TE cells

See Supplementary File 1

Supplementary Table 2: Correlation of FGF2, FGF10, FGFR1 and FGFR2 with a cancer marker (AMACR) and a stromal marker (FGF7) in primary tumors

	FGF2	FGF10	FGFR1	FGFR2
**AMACR**	-0.39	-0.38	-0.41	-0.37
**FGF7**	0.53	0.37	0.45	0.37
