# Supplementary material for: Fibroblast growth factor receptor signaling plays a key role in transformation induced by the TMPRSS2/ERG fusion gene and decreased PTEN
Source: Oncotarget. 2018 Feb 12;9(18):14456–71. doi: 10.18632/oncotarget.24470 (PMC5865682; doi:10.18632/oncotarget.24470)
Supplement: Supplementary file 2 [file oncotarget-09-14456-s002.docx]

|  | **Supplementary Table 1. Protein coding genes altered in only the PTEN KD/TE cells** |  |
| --- | --- | --- |
| **Gene Symbol** | **Gene Name** | **Fold** |
| ABCA7 | ref\|Homo sapiens ATP-binding cassette, sub-family A (ABC1), member 7 (ABCA7), mRNA [NM_019112] | 1.6970852 |
| ABI3BP | ref\|Homo sapiens ABI family, member 3 (NESH) binding protein (ABI3BP), mRNA [NM_015429] | 3.338516 |
| ADAM33 | ref\|Homo sapiens ADAM metallopeptidase domain 33 (ADAM33), transcript variant 2, mRNA [NM_153202] | 1.5899361 |
| ADAMTS7 | ref\|Homo sapiens ADAM metallopeptidase with thrombospondin type 1 motif, 7 (ADAMTS7), mRNA [NM_014272] | 1.8742313 |
| ADAMTSL5 | ref\|Homo sapiens ADAMTS-like 5 (ADAMTSL5), mRNA [NM_213604] | 1.479462 |
| ANKRD20A1 | ref\|Homo sapiens ankyrin repeat domain 20 family, member A1 (ANKRD20A1), mRNA [NM_032250] | 1.9808942 |
| APOL4 | ref\|Homo sapiens apolipoprotein L, 4 (APOL4), transcript variant a, mRNA [NM_030643] | 2.2176784 |
| ARL17 | ref\|Homo sapiens ADP-ribosylation factor-like 17 (ARL17), transcript variant 1, mRNA [NM_001039083] | 2.1057629 |
| ARL2 | ref\|Homo sapiens ADP-ribosylation factor-like 2 (ARL2), mRNA [NM_001667] | 1.722978 |
| ASPSCR1 | ref\|Homo sapiens alveolar soft part sarcoma chromosome region, candidate 1 (ASPSCR1), mRNA [NM_024083] | 1.9887325 |
| ATPIF1 | ref\|Homo sapiens ATPase inhibitory factor 1 (ATPIF1), nuclear gene encoding mitochondrial protein, transcript variant 1, mRNA | 2.2631674 |
| B4GALNT4 | ref\|Homo sapiens beta-1,4-N-acetyl-galactosaminyl transferase 4 (B4GALNT4), mRNA [NM_178537] | 1.8883985 |
| B4GALT2 | ref\|Homo sapiens UDP-Gal:betaGlcNAc beta 1,4- galactosyltransferase, polypeptide 2 (B4GALT2), transcript variant 2 | 2.4627972 |
| BAT3 | ref\|Homo sapiens HLA-B associated transcript 3 (BAT3), transcript variant 1, mRNA [NM_004639] | 0.5272955 |
| BC031259 | gb\|Homo sapiens ELK2, member of ETS oncogene family, pseudogene 1, mRNA (cDNA clone IMAGE:5272595). [BC031259] | 1.4637152 |
| BCAN | ref\|Homo sapiens brevican (BCAN), transcript variant 2, mRNA [NM_198427] | 1.7434201 |
| BCL7A | ref\|Homo sapiens B-cell CLL/lymphoma 7A (BCL7A), transcript variant 1, mRNA [NM_020993] | 0.6790219 |
| BHLHA9 | ref\|Homo sapiens basic helix-loop-helix family, member a9 (BHLHA9), mRNA [NM_001164405] | 2.3313026 |
| BHLHE23 | ref\|Homo sapiens basic helix-loop-helix family, member e23 (BHLHE23), mRNA [NM_080606] | 1.7936237 |
| BX538057 | gb\|Homo sapiens mRNA; cDNA DKFZp686J1595 (from clone DKFZp686J1595) [BX538057] | 0.5234455 |
| C10orf47 | ref\|Homo sapiens chromosome 10 open reading frame 47 (C10orf47), mRNA [NM_153256] | 1.6479776 |
| C11orf95 | ref\|Homo sapiens chromosome 11 open reading frame 95 (C11orf95), mRNA [NM_001144936] | 1.4275201 |
| C14orf104 | ref\|Homo sapiens chromosome 14 open reading frame 104 (C14orf104), transcript variant 2, mRNA [NM_001083908] | 2.0452106 |
| C18orf23 | gb\|Homo sapiens chromosome 18 open reading frame 23, mRNA (cDNA clone MGC:120885 IMAGE:7939695) | 1.5342625 |
| C19orf25 | ref\|Homo sapiens chromosome 19 open reading frame 25 (C19orf25), mRNA [NM_152482] | 1.8298508 |
| C19orf44 | ref\|Homo sapiens chromosome 19 open reading frame 44 (C19orf44), mRNA [NM_032207] | 1.697904 |
| C19orf60 | ref\|Homo sapiens chromosome 19 open reading frame 60 (C19orf60), transcript variant 1, mRNA [NM_001100418] | 1.7639723 |
| C1orf226 | ref\|Homo sapiens chromosome 1 open reading frame 226 (C1orf226), transcript variant 2, mRNA [NM_001085375] | 1.5768048 |
| C1orf229 | ref\|Homo sapiens chromosome 1 open reading frame 229 (C1orf229), mRNA [NM_207401] | 1.5141744 |
| C1orf86 | ref\|Homo sapiens chromosome 1 open reading frame 86 (C1orf86), transcript variant 2, mRNA [NM_182533] | 1.9283765 |
| C4orf48 | ref\|Homo sapiens chromosome 4 open reading frame 48 (C4orf48), mRNA [NM_001141936] | 1.9546793 |
| C5orf43 | ref\|Homo sapiens chromosome 5 open reading frame 43 (C5orf43), mRNA [NM_001048249] | 1.8158416 |
| C6orf204 | ref\|Homo sapiens chromosome 6 open reading frame 204 (C6orf204), transcript variant 2, mRNA [NM_206921] | 0.5869264 |
| C6orf48 | ref\|Homo sapiens chromosome 6 open reading frame 48 (C6orf48), transcript variant 1, mRNA [NM_001040437] | 2.1236168 |
| C7orf41 | ref\|Homo sapiens chromosome 7 open reading frame 41 (C7orf41), mRNA [NM_152793] | 1.5529707 |
| CACNA2D3 | ref\|Homo sapiens calcium channel, voltage-dependent, alpha 2/delta subunit 3 (CACNA2D3), mRNA [NM_018398] | 1.4870335 |
| CAST | ref\|Homo sapiens calpastatin (CAST), transcript variant 1, mRNA [NM_001750] | 0.5618239 |
| CC2D1B | ref\|Homo sapiens coiled-coil and C2 domain containing 1B (CC2D1B), mRNA [NM_032449] | 1.6362465 |
| CCDC55 | ref\|Homo sapiens coiled-coil domain containing 55 (CCDC55), transcript variant 1, mRNA [NM_032141] | 2.1595091 |
| CCL2 | ref\|Homo sapiens chemokine (C-C motif) ligand 2 (CCL2), mRNA [NM_002982] | 0.1881182 |
| CCNB3 | ref\|Homo sapiens cyclin B3 (CCNB3), transcript variant 3, mRNA [NM_033031] | 1.9509019 |
| CD276 | ref\|Homo sapiens CD276 molecule (CD276), transcript variant 1, mRNA [NM_001024736] | 1.7973464 |
| CD7 | ref\|Homo sapiens CD7 molecule (CD7), mRNA [NM_006137] | 1.8002513 |
| CDT1 | ref\|Homo sapiens chromatin licensing and DNA replication factor 1 (CDT1), mRNA [NM_030928] | 0.5972269 |
| CDV3 | ref\|Homo sapiens CDV3 homolog (mouse) (CDV3), transcript variant 2, mRNA [NM_017548] | 1.6475559 |
| CECR2 | ref\|Homo sapiens cat eye syndrome chromosome region, candidate 2 (CECR2), mRNA [NM_031413] | 0.6681512 |
| CEP76 | ref\|Homo sapiens centrosomal protein 76kDa (CEP76), mRNA [NM_024899] | 0.595124 |
| CHD1 | ref\|Homo sapiens chromodomain helicase DNA binding protein 1 (CHD1), mRNA [NM_001270] | 0.4809607 |
| CIT | ref\|Homo sapiens citron (rho-interacting, serine/threonine kinase 21) (CIT), mRNA [NM_007174] | 0.5588103 |
| CITED1 | ref\|Homo sapiens Cbp/p300-interacting transactivator, with Glu/Asp-rich carboxy-terminal domain, 1 | 1.9644313 |
| CLDN9 | ref\|Homo sapiens claudin 9 (CLDN9), mRNA [NM_020982] | 2.1539666 |
| CLPTM1L | ref\|Homo sapiens CLPTM1-like (CLPTM1L), mRNA [NM_030782] | 1.4508591 |
| CMTM5 | ref\|Homo sapiens CKLF-like MARVEL transmembrane domain containing 5 (CMTM5), transcript variant 3, mRNA [NM_001037288] | 1.9904925 |
| CNTN4 | ref\|Homo sapiens contactin 4 (CNTN4), transcript variant 3, mRNA [NM_175613] | 1.8076973 |
| COL6A6 | ref\|Homo sapiens collagen type VI alpha 6 (COL6A6), mRNA [NM_001102608] | 0.6158447 |
| CP110 | ref\|Homo sapiens CP110 protein (CP110), mRNA [NM_014711] | 0.6015712 |
| CRELD2 | ref\|Homo sapiens cysteine-rich with EGF-like domains 2 (CRELD2), transcript variant 2, mRNA [NM_024324] | 1.6634011 |
| CRIP1 | ref\|Homo sapiens cysteine-rich protein 1 (intestinal) (CRIP1), mRNA [NM_001311] | 1.8756016 |
| CRTC1 | ref\|Homo sapiens CREB regulated transcription coactivator 1 (CRTC1), transcript variant 3, mRNA [NM_001098482] | 1.675237 |
| CSH2 | ref\|Homo sapiens chorionic somatomammotropin hormone 2 (CSH2), transcript variant 2, mRNA [NM_022644] | 1.7131511 |
| CTAG1A | ref\|Homo sapiens cancer/testis antigen 1A (CTAG1A), mRNA [NM_139250] | 1.7516796 |
| CTBP1 | ref\|Homo sapiens C-terminal binding protein 1 (CTBP1), transcript variant 2, mRNA [NM_001012614] | 0.4846556 |
| CTNNBIP1 | ref\|Homo sapiens catenin, beta interacting protein 1 (CTNNBIP1), transcript variant 1, mRNA [NM_020248] | 2.0283023 |
| DB028998 | gb\|DB028998 TESTI2 Homo sapiens cDNA clone TESTI2012045 5', mRNA sequence [DB028998] | 1.5536544 |
| DB229023 | gb\|DB229023 TRACH3 Homo sapiens cDNA clone TRACH3022015 5', mRNA sequence [DB229023] | 1.6312996 |
| DBH | ref\|Homo sapiens dopamine beta-hydroxylase (dopamine beta-monooxygenase) (DBH), mRNA [NM_000787] | 1.7265724 |
| DBX1 | ref\|Homo sapiens developing brain homeobox 1 (DBX1), mRNA [NM_001029865] | 1.4356355 |
| DDX49 | ref\|Homo sapiens DEAD (Asp-Glu-Ala-Asp) box polypeptide 49 (DDX49), mRNA [NM_019070] | 1.4952471 |
| DEFA4 | ref\|Homo sapiens defensin, alpha 4, corticostatin (DEFA4), mRNA [NM_001925] | 0.6518775 |
| DENND3 | ref\|Homo sapiens DENN/MADD domain containing 3 (DENND3), mRNA [NM_014957] | 1.6592644 |
| DFFB | ref\|Homo sapiens DNA fragmentation factor, 40kDa, beta polypeptide (caspase-activated DNase) (DFFB), mRNA [NM_004402] | 2.1069548 |
| DHX34 | ref\|Homo sapiens DEAH (Asp-Glu-Ala-His) box polypeptide 34 (DHX34), mRNA [NM_014681] | 3.8228515 |
| DLK1 | ref\|Homo sapiens delta-like 1 homolog (Drosophila) (DLK1), mRNA [NM_003836] | 1.6510351 |
| DPM2 | ref\|Homo sapiens dolichyl-phosphate mannosyltransferase polypeptide 2, regulatory subunit (DPM2), mRNA [NM_003863] | 0.6388808 |
| DRAM1 | ref\|Homo sapiens DNA-damage regulated autophagy modulator 1 (DRAM1), mRNA [NM_018370] | 0.5540144 |
| DUSP15 | ref\|Homo sapiens dual specificity phosphatase 15 (DUSP15), transcript variant 1, mRNA [NM_080611] | 3.2665082 |
| DUX4 | ref\|Homo sapiens double homeobox, 4 (DUX4), mRNA [NM_033178] | 1.8654154 |
| DYRK1B | ref\|Homo sapiens dual-specificity tyrosine-(Y)-phosphorylation regulated kinase 1B (DYRK1B), transcript variant a, mRNA [NM_004714] | 1.9704326 |
| ECHDC3 | ref\|Homo sapiens enoyl Coenzyme A hydratase domain containing 3 (ECHDC3), nuclear gene encoding mitochondrial protein, mRNA | 2.1914679 |
| EDN1 | ref\|Homo sapiens endothelin 1 (EDN1), mRNA [NM_001955] | 0.584268 |
| EMILIN1 | ref\|Homo sapiens elastin microfibril interfacer 1 (EMILIN1), mRNA [NM_007046] | 1.6671682 |
| ENST00000238576 | ens\|Putative alpha-1-antitrypsin-related protein Precursor | 1.9083419 |
| ENST00000249399 | ens\|Putative uncharacterized protein ENSP00000249399 [Source:UniProtKB/TrEMBL;Acc:B7WPQ3] [ENST00000249399] | 1.6985627 |
| ENST00000288766 | ens\|1-phosphatidylinositol-4,5-bisphosphate phosphodiesterase eta-2 (EC 3.1.4.1(Phosphoinositide phospholipase C-like 4) | 1.8741503 |
| ENST00000314963 | ens\|Putative uncharacterized protein LOC439951 [Source:UniProtKB/Swiss-Prot;Acc:Q8NDZ9] [ENST00000314963] | 2.1795251 |
| ENST00000320778 | ens\|Putative uncharacterized protein C9orf62 [Source:UniProtKB/Swiss-Prot;Acc:Q8N4C0] [ENST00000320778] | 1.497276 |
| ENST00000323800 | ens\|CDNA FLJ25155 fis, clone CBR07976 [Source:UniProtKB/TrEMBL;Acc:Q96LR6] [ENST00000323800] | 1.6141491 |
| ENST00000329504 | ens\|Kinesin-like protein KIF26B | 2.3304101 |
| ENST00000342518 | ens\|D-2-hydroxyglutarate dehydrogenase, mitochondrial Precursor (EC 1.1.99.-) | 1.575516 |
| ENST00000368950 | ens\|Adenylate kinase domain-containing protein 2 | 2.1970525 |
| ENST00000369123 | ens\|Chromosome 6 open reading frame 220 Fragment | 1.8943764 |
| ENST00000371623 | ens\|Prostaglandin-H2 D-isomerase Precursor (EC 5.3.99.2)(Lipocalin-type prostaglandin-D synthase)(Glutathione-independent PGD synthetase) | 1.8250027 |
| ENST00000376536 | ens\|Keratin-associated protein 5-10 (Keratin-associated protein 5.10)(Ultrahigh sulfur keratin-associated protein 5.10) | 1.6238459 |
| ENST00000378563 | ens\|Protein kinase C zeta type (EC 2.7.11.13)(nPKC-zeta) [Source:UniProtKB/Swiss-Prot;Acc:Q05513] [ENST00000378563] | 1.5440897 |
| ENST00000389653 | ens\|Protein Dok-7 (Downstream of tyrosine kinase 7) | 2.5782754 |
| ENST00000394478 | ens\|Cohesin subunit SA-2 (Stromal antigen 2)(SCC3 homolog 2) [Source:UniProtKB/Swiss-Prot;Acc:Q8N3U4] | 0.5712852 |
| ENST00000396017 | ens\|Potassium voltage-gated channel subfamily G member 1 (Voltage-gated potassium channel subunit Kv6.1)(kH2) | 0.6394441 |
| ENST00000396513 | ens\|Putative uncharacterized protein ENSP00000379770 Fragment [Source:UniProtKB/TrEMBL;Acc:A8MXN8] [ENST00000396513] | 2.067636 |
| ENST00000396532 | ens\|Zinc finger CCCH domain-containing protein 7B (Rotavirus 'X'-associated non-structural protein) | 2.5260463 |
| ENST00000398726 | ens\|Putative uncharacterized protein ENSP00000381711 Fragment [Source:UniProtKB/TrEMBL;Acc:A8MYH6] [ENST00000398726] | 1.4770221 |
| ENST00000403506 | ens\|Uncharacterized protein C2orf63 [Source:UniProtKB/Swiss-Prot;Acc:Q8NHS4] [ENST00000403506] | 1.9959513 |
| ENST00000420834 | ens\|Putative uncharacterized protein C19orf31 [Source:UniProtKB/Swiss-Prot;Acc:Q8N1I2] [ENST00000420834] | 1.8536028 |
| ENST00000421059 | ens\|NADPH--cytochrome P450 reductase (CPR)(P450R)(EC 1.6.2.4) | 2.9419076 |
| ENST00000447142 | ens\|Putative uncharacterized protein ENSP00000341218 | 1.9590562 |
| ENST00000447753 | ens\|cDNA FLJ44869 fis, clone BRAMY2015516 [Source:UniProtKB/TrEMBL;Acc:Q6ZQT2] [ENST00000447753] | 1.7590631 |
| EPB41L1 | ref\|Homo sapiens erythrocyte membrane protein band 4.1-like 1 (EPB41L1), transcript variant 2, mRNA [NM_177996] | 2.1032616 |
| ESRRA | ref\|Homo sapiens estrogen-related receptor alpha (ESRRA), mRNA [NM_004451] | 1.5341745 |
| EWSR1 | ref\|Homo sapiens Ewing sarcoma breakpoint region 1 (EWSR1), transcript variant 1, mRNA [NM_013986] | 0.5959195 |
| FABP1 | ref\|Homo sapiens fatty acid binding protein 1, liver (FABP1), mRNA [NM_001443] | 1.9256158 |
| FAHD1 | ref\|Homo sapiens fumarylacetoacetate hydrolase domain containing 1 (FAHD1), nuclear gene encoding mitochondrial protein, transcript variant 1 | 0.5746982 |
| FAM129C | ref\|Homo sapiens family with sequence similarity 129, member C (FAM129C), transcript variant 2, mRNA [NM_001098524] | 1.707275 |
| FAM169A | ref\|Homo sapiens family with sequence similarity 169, member A (FAM169A), mRNA [NM_015566] | 0.6278805 |
| FAM192A | ref\|Homo sapiens family with sequence similarity 192, member A (FAM192A), mRNA [NM_024946] | 0.6142007 |
| FAM22F | ref\|Homo sapiens family with sequence similarity 22, member F (FAM22F), mRNA [NM_017561] | 2.0926778 |
| FAM82A1 | ref\|Homo sapiens family with sequence similarity 82, member A1 (FAM82A1), mRNA [NM_144713] | 0.5378473 |
| FBRSL1 | ref\|Homo sapiens fibrosin-like 1 (FBRSL1), mRNA [NM_001142641] | 1.8306848 |
| FERD3L | ref\|Homo sapiens Fer3-like (Drosophila) (FERD3L), mRNA [NM_152898] | 1.6403195 |
| FGF3 | ref\|Homo sapiens fibroblast growth factor 3 (murine mammary tumor virus integration site (v-int-2) oncogene homolog) (FGF3), mRNA | 1.5016896 |
| FGFR4 | ref\|Homo sapiens fibroblast growth factor receptor 4 (FGFR4), transcript variant 3, mRNA [NM_213647] | 2.0974112 |
| FLI1 | ref\|Homo sapiens Friend leukemia virus integration 1 (FLI1), mRNA [NM_002017] | 0.5475996 |
| FLJ35220 | ref\|Homo sapiens hypothetical protein FLJ35220 (FLJ35220), transcript variant 1, mRNA [NM_173627] | 2.1853953 |
| FLJ42392 | gb\|Homo sapiens cDNA FLJ42392 fis, clone ACTVT2000380 [AK124383] | 1.5990525 |
| FLJ43879 | ref\|PREDICTED: Homo sapiens FLJ43879 protein (FLJ43879), miscRNA [XR_041560] | 1.7277134 |
| FLJ44082 | ref\|Homo sapiens FAM75-like protein FLJ44082 (FLJ44082), mRNA [NM_207416] | 1.6033362 |
| FOXC2 | ref\|Homo sapiens forkhead box C2 (MFH-1, mesenchyme forkhead 1) (FOXC2), mRNA [NM_005251] | 1.7464346 |
| FOXP4 | ref\|Homo sapiens forkhead box P4 (FOXP4), transcript variant 1, mRNA [NM_001012426] | 1.7371279 |
| FRY | ref\|Homo sapiens furry homolog (Drosophila) (FRY), mRNA [NM_023037] | 1.8816507 |
| GALK1 | ref\|Homo sapiens galactokinase 1 (GALK1), mRNA [NM_000154] | 2.1018397 |
| GATAD2A | ref\|Homo sapiens GATA zinc finger domain containing 2A (GATAD2A), mRNA [NM_017660] | 0.5283504 |
| GNAO1 | ref\|Homo sapiens guanine nucleotide binding protein (G protein), alpha activating activity polypeptide O (GNAO1), transcript variant 2, mRNA | 1.7710854 |
| GNAS | ref\|Homo sapiens GNAS complex locus (GNAS), transcript variant 4, mRNA [NM_016592] | 1.8685969 |
| GOLGA2LY1 | ref\|Homo sapiens golgi autoantigen, golgin subfamily a, 2-like, Y-linked 1 (GOLGA2LY1), non-coding RNA [NR_001555] | 1.6762124 |
| GOLGA6L6 | ref\|Homo sapiens golgi autoantigen, golgin subfamily a, 6-like 6 (GOLGA6L6), mRNA [NM_001145004] | 2.0212518 |
| GPR149 | ref\|Homo sapiens G protein-coupled receptor 149 (GPR149), mRNA | 2.4169222 |
| GPR150 | ref\|Homo sapiens G protein-coupled receptor 150 (GPR150), mRNA [NM_199243] | 2.2346745 |
| GPR25 | ref\|Homo sapiens G protein-coupled receptor 25 (GPR25), mRNA [NM_005298] | 1.6338998 |
| GSC2 | ref\|Homo sapiens goosecoid homeobox 2 (GSC2), mRNA [NM_005315] | 1.7006116 |
| HACE1 | ref\|Homo sapiens HECT domain and ankyrin repeat containing, E3 ubiquitin protein ligase 1 (HACE1), mRNA [NM_020771] | 1.5019496 |
| HBG1 | ref\|Homo sapiens hemoglobin, gamma A (HBG1), mRNA [NM_000559] | 1.628769 |
| hCG_2003663 | gb\|Homo sapiens cDNA: FLJ21498 fis, clone COL05627 [AK025151] | 1.7451098 |
| HIPK2 | ref\|Homo sapiens homeodomain interacting protein kinase 2 (HIPK2), transcript variant 2, mRNA [NM_001113239] | 2.1315695 |
| HIST2H2AB | ref\|Homo sapiens histone cluster 2, H2ab (HIST2H2AB), mRNA [NM_175065] | 1.9663348 |
| HIST2H2AC | ref\|Homo sapiens histone cluster 2, H2ac (HIST2H2AC), mRNA [NM_003517] | 0.5880405 |
| HIST2H2BF | ref\|Homo sapiens histone cluster 2, H2bf (HIST2H2BF), transcript variant 2, mRNA [NM_001161334] | 1.7433498 |
| HIST2H3D | ref\|Homo sapiens histone cluster 2, H3d (HIST2H3D), mRNA [NM_001123375] | 1.5152235 |
| HMG20B | ref\|Homo sapiens high-mobility group 20B (HMG20B), mRNA [NM_006339] | 1.6694572 |
| HMGA2 | ref\|Homo sapiens high mobility group AT-hook 2 (HMGA2), transcript variant 2, mRNA [NM_003484] | 1.6815309 |
| HMX1 | ref\|Homo sapiens H6 family homeobox 1 (HMX1), mRNA [NM_018942] | 1.9296276 |
| HOXA10 | ref\|Homo sapiens homeobox A10 (HOXA10), transcript variant 1, mRNA [NM_018951] | 1.6749383 |
| HOXA7 | ref\|Homo sapiens homeobox A7 (HOXA7), mRNA [NM_006896] | 0.6407632 |
| HTR3A | ref\|Homo sapiens 5-hydroxytryptamine (serotonin) receptor 3A (HTR3A), transcript variant 1, mRNA [NM_213621] | 1.8880608 |
| IER2 | ref\|Homo sapiens immediate early response 2 (IER2), mRNA [NM_004907] | 0.6350295 |
| IER5L | ref\|Homo sapiens immediate early response 5-like (IER5L), mRNA [NM_203434] | 1.8039066 |
| ING3 | ref\|Homo sapiens inhibitor of growth family, member 3 (ING3), transcript variant 1, mRNA [NM_019071] | 0.5183671 |
| INTS1 | ref\|Homo sapiens integrator complex subunit 1 (INTS1), mRNA [NM_001080453] | 1.6826595 |
| IP6K1 | ref\|Homo sapiens inositol hexakisphosphate kinase 1 (IP6K1), transcript variant 1, mRNA [NM_153273] | 1.8554033 |
| ISYNA1 | ref\|Homo sapiens inositol-3-phosphate synthase 1 (ISYNA1), mRNA [NM_016368] | 1.8259452 |
| ITFG2 | ref\|Homo sapiens integrin alpha FG-GAP repeat containing 2 (ITFG2), mRNA [NM_018463] | 0.6451315 |
| ITPA | ref\|Homo sapiens inosine triphosphatase (nucleoside triphosphate pyrophosphatase) (ITPA), transcript variant 1, mRNA [NM_033453] | 2.0730814 |
| IVL | ref\|Homo sapiens involucrin (IVL), mRNA [NM_005547] | 1.7139534 |
| JSRP1 | ref\|Homo sapiens junctional sarcoplasmic reticulum protein 1 (JSRP1), mRNA [NM_144616] | 2.422756 |
| JUND | ref\|Homo sapiens jun D proto-oncogene (JUND), mRNA [NM_005354] | 2.6147168 |
| KIAA1210 | ref\|Homo sapiens KIAA1210 (KIAA1210), mRNA [NM_020721] | 0.6243569 |
| KIAA1704 | ref\|Homo sapiens KIAA1704 (KIAA1704), mRNA [NM_018559] | 0.6563768 |
| KIFC1 | ref\|Homo sapiens kinesin family member C1 (KIFC1), mRNA [NM_002263] | 0.5951116 |
| KLF16 | ref\|Homo sapiens Kruppel-like factor 16 (KLF16), mRNA [NM_031918] | 1.4856822 |
| KLF2 | ref\|Homo sapiens Kruppel-like factor 2 (lung) (KLF2), mRNA [NM_016270] | 0.4766921 |
| KLHL34 | ref\|Homo sapiens kelch-like 34 (Drosophila) (KLHL34), mRNA [NM_153270] | 1.7231435 |
| KRT17 | ref\|Homo sapiens keratin 17 (KRT17), mRNA [NM_000422] | 1.9285993 |
| KRTAP3-1 | ref\|Homo sapiens keratin associated protein 3-1 (KRTAP3-1), mRNA [NM_031958] | 1.5337288 |
| LCE1A | ref\|Homo sapiens late cornified envelope 1A (LCE1A), mRNA [NM_178348] | 1.6321282 |
| LCE1D | ref\|Homo sapiens late cornified envelope 1D (LCE1D), mRNA [NM_178352] | 1.7655924 |
| LCE3B | ref\|Homo sapiens late cornified envelope 3B (LCE3B), mRNA [NM_178433] | 1.7394394 |
| LGALS7 | ref\|Homo sapiens lectin, galactoside-binding, soluble, 7 (LGALS7), mRNA [NM_002307] | 1.764602 |
| LGTN | ref\|Homo sapiens ligatin (LGTN), mRNA [NM_006893] | 0.5233273 |
| LHFPL5 | ref\|Homo sapiens lipoma HMGIC fusion partner-like 5 (LHFPL5), mRNA [NM_182548] | 1.7237015 |
| LOC100128142 | ref\|PREDICTED: Homo sapiens similar to RIKEN cDNA E130309D14 (LOC100128142), mRNA [XM_001715360] | 1.7536804 |
| LOC100128703 | ref\|PREDICTED: Homo sapiens hypothetical protein LOC100128703 (LOC100128703), mRNA [XM_002344534] | 1.8113206 |
| LOC100128825 | ref\|PREDICTED: Homo sapiens similar to aldose reductase (LOC100128825), miscRNA [XR_037650] | 1.5673142 |
| LOC100129722 | gb\|Homo sapiens cDNA FLJ40445 fis, clone TESTI2040297 [AK097764] | 1.8275749 |
| LOC100131009 | ref\|PREDICTED: Homo sapiens hypothetical LOC100131009 (LOC100131009), mRNA [XM_001714966] | 1.7655726 |
| LOC100131244 | ref\|PREDICTED: Homo sapiens similar to kinase D-interacting substrate 220 (LOC100131244), mRNA [XM_001719946] | 1.7176071 |
| LOC100131346 | gb\|Homo sapiens cDNA FLJ45704 fis, clone FEBRA2026977. [AK127606] | 1.6036742 |
| LOC100132168 | ref\|PREDICTED: Homo sapiens hypothetical LOC100132168 (LOC100132168), mRNA [XM_001717089] | 1.8097556 |
| LOC100132515 | ref\|PREDICTED: Homo sapiens similar to hCG2045429 (LOC100132515), mRNA [XM_001719495] | 3.0750262 |
| LOC100132848 | ref\|PREDICTED: Homo sapiens hypothetical LOC100132848 (LOC100132848), mRNA [XM_001715611] | 2.2708801 |
| LOC100134002 | ref\|PREDICTED: Homo sapiens hypothetical LOC100134002 (LOC100134002), mRNA [XM_001714782] | 1.5589522 |
| LOC100134702 | ref\|PREDICTED: Homo sapiens similar to mucin (LOC100134702), mRNA [XM_002344283] | 1.9424697 |
| LOC100291206 | ref\|PREDICTED: Homo sapiens hypothetical protein LOC100291206 (LOC100291206), mRNA [XM_002346973] | 4.1004695 |
| LOC100292717 | ref\|PREDICTED: Homo sapiens similar to arg tyrosine kinase (LOC100292717), mRNA [XM_002345084] | 1.8430574 |
| LOC169834 | ref\|Homo sapiens hypothetical protein LOC169834 (LOC169834), mRNA [NM_001101338] | 0.6384513 |
| LOC285740 | gb\|AGENCOURT_6565309 NIH_MGC_119 Homo sapiens cDNA clone IMAGE:5744234 5', mRNA sequence [BM559483] | 2.1209911 |
| LOC390298 | ref\|PREDICTED: Homo sapiens similar to translocase of inner mitochondrial membrane 17 homolog B (LOC390298), miscRNA | 2.0293544 |
| LOC402509 | ref\|PREDICTED: Homo sapiens similar to solute carrier family 29 (nucleoside transporters), member 4 (LOC402509), mRNA | 2.0537029 |
| LOC441601 | ref\|Homo sapiens septin 7 pseudogene (LOC441601), non-coding RNA [NR_003034] | 1.6215431 |
| LOC442283 | ref\|PREDICTED: Homo sapiens similar to zinc finger protein 717 (LOC442283), miscRNA [XR_042501] | 1.885817 |
| LOC645937 | ref\|PREDICTED: Homo sapiens similar to zinc finger protein 598 (LOC645937), mRNA [XM_928905] | 0.5680849 |
| LOC652119 | ref\|PREDICTED: Homo sapiens similar to putative DUX4 protein (LOC652119), mRNA [XM_001720798] | 1.5708481 |
| LOC727804 | ref\|PREDICTED: Homo sapiens hypothetical LOC727804 (LOC727804), mRNA [XM_001125843] | 1.524724 |
| LOC728846 | ref\|PREDICTED: Homo sapiens hypothetical LOC728846 (LOC728846), mRNA [XM_001715013] | 1.6007936 |
| LOC729978 | ens\|NPIP-like protein 2 [Source:UniProtKB/Swiss-Prot;Acc:A6NJ64] [ENST00000399147] | 0.5908238 |
| LOC729986 | ref\|PREDICTED: Homo sapiens hypothetical LOC729986 (LOC729986), mRNA [XM_001131545] | 1.9002504 |
| LOC730098 | ref\|PREDICTED: Homo sapiens similar to chemokine (C-C motif) ligand 27 (LOC730098), miscRNA [XR_041278] | 1.4450831 |
| LOC730375 | ref\|PREDICTED: Homo sapiens hypothetical LOC730375 (LOC730375), miscRNA [XR_039647] | 0.4386793 |
| LONP1 | ref\|Homo sapiens lon peptidase 1, mitochondrial (LONP1), nuclear gene encoding mitochondrial protein, mRNA [NM_004793] | 2.0185234 |
| LPCAT1 | ref\|Homo sapiens lysophosphatidylcholine acyltransferase 1 (LPCAT1), mRNA [NM_024830] | 1.8206907 |
| LPCAT2 | ref\|Homo sapiens lysophosphatidylcholine acyltransferase 2 (LPCAT2), mRNA [NM_017839] | 0.5403244 |
| LRRC1 | gb\|AU119761 HEMBA1 Homo sapiens cDNA clone HEMBA1006583 5', mRNA sequence [AU119761] | 1.950885 |
| LSM14B | ref\|Homo sapiens LSM14B, SCD6 homolog B (S. cerevisiae) (LSM14B), mRNA [NM_144703] | 0.5374694 |
| LTB4R2 | ref\|Homo sapiens leukotriene B4 receptor 2 (LTB4R2), mRNA [NM_019839] | 1.8332908 |
| LTV1 | ref\|Homo sapiens LTV1 homolog (S. cerevisiae) (LTV1), mRNA [NM_032860] | 1.5619613 |
| LUC7L | ref\|Homo sapiens LUC7-like (S. cerevisiae) (LUC7L), transcript variant 2, mRNA [NM_201412] | 0.5968595 |
| MAGEA11 | ref\|Homo sapiens melanoma antigen family A, 11 (MAGEA11), transcript variant 2, mRNA [NM_001011544] | 2.0053561 |
| MANEAL | ref\|Homo sapiens mannosidase, endo-alpha-like (MANEAL), transcript variant 1, mRNA [NM_001031740] | 1.9795304 |
| MAP2K7 | ref\|Homo sapiens mitogen-activated protein kinase kinase 7 (MAP2K7), mRNA [NM_145185] | 0.594602 |
| MAP7D3 | ref\|Homo sapiens MAP7 domain containing 3 (MAP7D3), mRNA [NM_024597] | 1.6564292 |
| MAPK13 | ref\|Homo sapiens mitogen-activated protein kinase 13 (MAPK13), mRNA [NM_002754] | 1.5009325 |
| MCHR2 | ref\|Homo sapiens melanin-concentrating hormone receptor 2 (MCHR2), transcript variant 1, mRNA [NM_001040179] | 1.961245 |
| MDM4 | ref\|Homo sapiens Mdm4 p53 binding protein homolog (mouse) (MDM4), transcript variant 1, mRNA [NM_002393] | 0.6516186 |
| MEX3D | ref\|Homo sapiens mex-3 homolog D (C. elegans) (MEX3D), mRNA [NM_203304] | 3.6051243 |
| MGAT5B | ref\|Homo sapiens mannosyl (alpha-1,6-)-glycoprotein beta-1,6-N-acetyl-glucosaminyltransferase, isozyme B (MGAT5B), transcript variant 1, mRNA | 1.8157612 |
| MLL4 | ref\|Homo sapiens myeloid/lymphoid or mixed-lineage leukemia 4 (MLL4), mRNA [NM_014727] | 1.673707 |
| MMP17 | ref\|Homo sapiens matrix metallopeptidase 17 (membrane-inserted) (MMP17), mRNA [NM_016155] | 1.6376982 |
| MORN3 | ref\|Homo sapiens MORN repeat containing 3 (MORN3), mRNA [NM_173855] | 1.6257451 |
| MRPS25 | ref\|Homo sapiens mitochondrial ribosomal protein S25 (MRPS25), nuclear gene encoding mitochondrial protein, mRNA [NM_022497] | 1.925994 |
| MTCH2 | ref\|Homo sapiens mitochondrial carrier homolog 2 (C. elegans) (MTCH2), nuclear gene encoding mitochondrial protein, mRNA | 1.7167733 |
| MVK | ref\|Homo sapiens mevalonate kinase (MVK), transcript variant 1, mRNA [NM_000431] | 1.5956505 |
| MYOT | ref\|Homo sapiens myotilin (MYOT), transcript variant 1, mRNA [NM_006790] | 1.7932287 |
| NACC1 | ref\|Homo sapiens nucleus accumbens associated 1, BEN and BTB (POZ) domain containing (NACC1), mRNA [NM_052876] | 0.3988434 |
| NAMPT | ref\|Homo sapiens nicotinamide phosphoribosyltransferase (NAMPT), mRNA [NM_005746] | 0.5477955 |
| NBPF1 | ref\|Homo sapiens neuroblastoma breakpoint family, member 1 (NBPF1), mRNA [NM_017940] | 0.5979195 |
| NCF1 | ref\|Homo sapiens neutrophil cytosolic factor 1 (NCF1), mRNA [NM_000265] | 2.8901435 |
| NFKBIL2 | ref\|Homo sapiens nuclear factor of kappa light polypeptide gene enhancer in B-cells inhibitor-like 2 (NFKBIL2), mRNA [NM_013432] | 1.467195 |
| NKX1-2 | ref\|Homo sapiens NK1 homeobox 2 (NKX1-2), mRNA [NM_001146340] | 2.3926218 |
| NKX2-5 | ref\|Homo sapiens NK2 transcription factor related, locus 5 (Drosophila) (NKX2-5), transcript variant 2, mRNA [NM_001166175] | 2.0184799 |
| NP103607 | tc\|GB\|S78510.1\|AAD14284.1 human major histocompatibility complex [NP103607] | 1.5391131 |
| NSUN6 | ref\|Homo sapiens NOL1/NOP2/Sun domain family, member 6 (NSUN6), mRNA [NM_182543] | 1.7210004 |
| NT5C1B | ref\|Homo sapiens 5'-nucleotidase, cytosolic IB (NT5C1B), transcript variant 1, mRNA [NM_001002006] | 1.7361678 |
| NT5C3L | ref\|Homo sapiens 5'-nucleotidase, cytosolic III-like (NT5C3L), mRNA [NM_052935] | 2.0842412 |
| NUBP2 | ref\|Homo sapiens nucleotide binding protein 2 (MinD homolog, E. coli) (NUBP2), mRNA [NM_012225] | 1.6504602 |
| OBFC2B | ref\|Homo sapiens oligonucleotide/oligosaccharide-binding fold containing 2B (OBFC2B), mRNA [NM_024068] | 0.6524529 |
| PACSIN2 | ref\|Homo sapiens protein kinase C and casein kinase substrate in neurons 2 (PACSIN2), mRNA [NM_007229] | 1.8770367 |
| PAEP | ref\|Homo sapiens progestagen-associated endometrial protein (PAEP), transcript variant 2, mRNA [NM_002571] | 2.8950641 |
| PANX2 | ref\|Homo sapiens pannexin 2 (PANX2), transcript variant 1, mRNA [NM_052839] | 1.7118617 |
| PCGF6 | ref\|Homo sapiens polycomb group ring finger 6 (PCGF6), transcript variant 1, mRNA [NM_001011663] | 0.6314642 |
| PCSK1N | ref\|Homo sapiens proprotein convertase subtilisin/kexin type 1 inhibitor (PCSK1N), mRNA [NM_013271] | 1.7993173 |
| PDZD4 | ref\|Homo sapiens PDZ domain containing 4 (PDZD4), mRNA [NM_032512] | 0.6228062 |
| PGD | ref\|Homo sapiens phosphogluconate dehydrogenase (PGD), mRNA [NM_002631] | 0.613281 |
| PIF1 | ref\|Homo sapiens PIF1 5'-to-3' DNA helicase homolog (S. cerevisiae) (PIF1), mRNA [NM_025049] | 0.6250431 |
| PKIG | ref\|Homo sapiens protein kinase (cAMP-dependent, catalytic) inhibitor gamma (PKIG), transcript variant 1, mRNA [NM_181805] | 1.5126364 |
| PLAU | ref\|Homo sapiens plasminogen activator, urokinase (PLAU), transcript variant 1, mRNA [NM_002658] | 0.4565934 |
| POU3F3 | ref\|Homo sapiens POU class 3 homeobox 3 (POU3F3), mRNA [NM_006236] | 1.5212329 |
| PPARD | ref\|Homo sapiens peroxisome proliferator-activated receptor delta (PPARD), transcript variant 1, mRNA [NM_006238] | 0.5870989 |
| PQBP1 | ref\|Homo sapiens polyglutamine binding protein 1 (PQBP1), transcript variant 1, mRNA [NM_005710] | 1.7561255 |
| PRR25 | ref\|Homo sapiens proline rich 25 (PRR25), mRNA [NM_001013638] | 3.0461292 |
| PTH2 | ref\|Homo sapiens parathyroid hormone 2 (PTH2), mRNA [NM_178449] | 2.5499178 |
| PTMA | ref\|Homo sapiens prothymosin, alpha (PTMA), transcript variant 2, mRNA [NM_002823] | 1.4399429 |
| PTPN20A | ref\|Homo sapiens protein tyrosine phosphatase, non-receptor type 20A (PTPN20A), transcript variant 2, mRNA [NM_001042387] | 1.7388299 |
| PURG | ref\|Homo sapiens purine-rich element binding protein G (PURG), transcript variant B, mRNA [NM_001015508] | 1.7402906 |
| QTRT1 | ref\|Homo sapiens queuine tRNA-ribosyltransferase 1 (QTRT1), mRNA [NM_031209] | 1.7074176 |
| RAB11FIP1 | ref\|Homo sapiens RAB11 family interacting protein 1 (class I) (RAB11FIP1), transcript variant 1, mRNA [NM_025151] | 1.9648038 |
| RAB11FIP1 | ref\|Homo sapiens RAB11 family interacting protein 1 (class I) (RAB11FIP1), transcript variant 2, mRNA [NM_001002233] | 0.6230353 |
| RASSF7 | ref\|Homo sapiens Ras association (RalGDS/AF-6) domain family (N-terminal) member 7 (RASSF7), transcript variant 1, mRNA [NM_003475] | 1.9273651 |
| RBM10 | ref\|Homo sapiens RNA binding motif protein 10 (RBM10), transcript variant 1, mRNA [NM_005676] | 1.9048487 |
| RCVRN | ref\|Homo sapiens recoverin (RCVRN), mRNA [NM_002903] | 0.6674398 |
| RDH13 | ref\|Homo sapiens retinol dehydrogenase 13 (all-trans/9-cis) (RDH13), nuclear gene encoding mitochondrial protein, transcript variant 2, mRNA | 1.7775978 |
| REXO1L1 | ref\|Homo sapiens REX1, RNA exonuclease 1 homolog (S. cerevisiae)-like 1 (REXO1L1), mRNA [NM_172239] | 2.5730752 |
| RFXANK | ref\|Homo sapiens regulatory factor X-associated ankyrin-containing protein (RFXANK), transcript variant 1, mRNA [NM_003721] | 1.5480749 |
| RHBDD3 | ref\|Homo sapiens rhomboid domain containing 3 (RHBDD3), mRNA [NM_012265] | 1.4503557 |
| RHBDF1 | ref\|Homo sapiens rhomboid 5 homolog 1 (Drosophila) (RHBDF1), mRNA [NM_022450] | 0.5414785 |
| RHD | ref\|Homo sapiens Rh blood group, D antigen (RHD), transcript variant 1, mRNA [NM_016124] | 0.5931933 |
| RNF166 | ref\|Homo sapiens ring finger protein 166 (RNF166), mRNA [NM_178841] | 0.5726679 |
| ROR2 | ref\|Homo sapiens receptor tyrosine kinase-like orphan receptor 2 (ROR2), mRNA [NM_004560] | 0.5434776 |
| RP3-402G11.5 | ref\|Homo sapiens selenoprotein O (SELO), mRNA [NM_031454] | 1.8182269 |
| RPL10L | ref\|Homo sapiens ribosomal protein L10-like (RPL10L), mRNA [NM_080746] | 1.4478643 |
| RPL13A | ref\|Homo sapiens ribosomal protein L13a (RPL13A), mRNA [NM_012423] | 1.489473 |
| RPL22 | ref\|Homo sapiens ribosomal protein L22 (RPL22), mRNA [NM_000983] | 1.5161163 |
| SAA4 | ref\|Homo sapiens serum amyloid A4, constitutive (SAA4), mRNA [NM_006512] | 1.5210894 |
| SAPS2 | ref\|Homo sapiens SAPS domain family, member 2 (SAPS2), mRNA [NM_014678] | 0.5891748 |
| SAT1 | ref\|Homo sapiens spermidine/spermine N1-acetyltransferase 1 (SAT1), transcript variant 1, mRNA [NM_002970] | 0.5468392 |
| SAV1 | ref\|Homo sapiens salvador homolog 1 (Drosophila) (SAV1), mRNA [NM_021818] | 1.938376 |
| SBK2 | ref\|Homo sapiens SH3-binding domain kinase family, member 2 (SBK2), mRNA [NM_001101401] | 1.8238151 |
| SC4MOL | ref\|Homo sapiens sterol-C4-methyl oxidase-like (SC4MOL), transcript variant 1, mRNA [NM_006745] | 0.559887 |
| SCARF2 | ref\|Homo sapiens scavenger receptor class F, member 2 (SCARF2), transcript variant 1, mRNA [NM_153334] | 2.2792534 |
| SCGB3A1 | ref\|Homo sapiens secretoglobin, family 3A, member 1 (SCGB3A1), mRNA [NM_052863] | 1.6177534 |
| SCML1 | ref\|Homo sapiens sex comb on midleg-like 1 (Drosophila) (SCML1), transcript variant 1, mRNA [NM_001037540] | 0.6005224 |
| SCN1B | ref\|Homo sapiens sodium channel, voltage-gated, type I, beta (SCN1B), transcript variant b, mRNA [NM_199037] | 1.5551695 |
| SEMA4A | ref\|Homo sapiens sema domain, immunoglobulin domain (Ig), transmembrane domain (TM) and short cytoplasmic domain | 2.046599 |
| SF3A2 | ref\|Homo sapiens splicing factor 3a, subunit 2, 66kDa (SF3A2), mRNA [NM_007165] | 0.4742029 |
| SFRS18 | ref\|Homo sapiens splicing factor, arginine/serine-rich 18 (SFRS18), transcript variant 1, mRNA [NM_032870] | 0.6227367 |
| SFTA1P | gb\|AGENCOURT_10018270 NIH_MGC_142 Homo sapiens cDNA clone IMAGE:6495000 5', mRNA sequence [BU601128] | 1.7588136 |
| SHROOM1 | ref\|Homo sapiens shroom family member 1 (SHROOM1), mRNA [NM_133456] | 1.9425407 |
| SHROOM3 | ref\|Homo sapiens shroom family member 3 (SHROOM3), mRNA [NM_020859] | 1.6433514 |
| SLC10A2 | ref\|Homo sapiens solute carrier family 10 (sodium/bile acid cotransporter family), member 2 (SLC10A2), mRNA [NM_000452] | 1.6030584 |
| SLC13A2 | ref\|Homo sapiens solute carrier family 13 (sodium-dependent dicarboxylate transporter), member 2 (SLC13A2) | 2.4768861 |
| SLC22A18 | ref\|Homo sapiens solute carrier family 22, member 18 (SLC22A18), transcript variant 2, mRNA [NM_183233] | 2.1791556 |
| SLC22A23 | ref\|Homo sapiens solute carrier family 22, member 23 (SLC22A23), transcript variant 1, mRNA [NM_015482] | 2.2970374 |
| SLC2A6 | ref\|Homo sapiens solute carrier family 2 (facilitated glucose transporter), member 6 (SLC2A6), transcript variant 1, mRNA [NM_017585] | 0.62795 |
| SLFNL1 | ref\|Homo sapiens schlafen-like 1 (SLFNL1), mRNA [NM_144990] | 1.7091173 |
| SNAR-G1 | gb\|AGENCOURT_10224340 NIH_MGC_141 Homo sapiens cDNA clone IMAGE:6565454 5', mRNA sequence [BU536871] | 1.5881286 |
| SNORA73A | ref\|Homo sapiens small nucleolar RNA, H/ACA box 73A (SNORA73A), small nucleolar RNA [NR_002907] | 1.4864866 |
| SORBS2 | ref\|Homo sapiens sorbin and SH3 domain containing 2 (SORBS2), transcript variant 2, mRNA [NM_021069] | 2.4999925 |
| SP8 | ref\|Homo sapiens Sp8 transcription factor (SP8), transcript variant 2, mRNA [NM_198956] | 1.8534122 |
| SPATA2L | ref\|Homo sapiens spermatogenesis associated 2-like (SPATA2L), mRNA [NM_152339] | 1.8639126 |
| SPP1 | ref\|Homo sapiens secreted phosphoprotein 1 (SPP1), transcript variant 1, mRNA [NM_001040058] | 2.0033534 |
| SPSB1 | ref\|Homo sapiens splA/ryanodine receptor domain and SOCS box containing 1 (SPSB1), mRNA [NM_025106] | 2.2500949 |
| SRD5A2 | ref\|Homo sapiens steroid-5-alpha-reductase, alpha polypeptide 2 (3-oxo-5 alpha-steroid delta 4-dehydrogenase alpha 2) (SRD5A2), mRNA | 1.8864959 |
| SRRM3 | ref\|Homo sapiens serine/arginine repetitive matrix 3 (SRRM3), mRNA [NM_001110199] | 1.6979267 |
| STAG3 | ref\|Homo sapiens stromal antigen 3 (STAG3), mRNA [NM_012447] | 2.0573943 |
| TBC1D10B | ref\|Homo sapiens TBC1 domain family, member 10B (TBC1D10B), mRNA [NM_015527] | 2.6140393 |
| TDRD1 | ref\|Homo sapiens tudor domain containing 1 (TDRD1), mRNA [NM_198795] | 1.6146241 |
| TFDP3 | ref\|Homo sapiens transcription factor Dp family, member 3 (TFDP3), mRNA [NM_016521] | 0.6445466 |
| THSD1 | ref\|Homo sapiens thrombospondin, type I, domain containing 1 (THSD1), transcript variant 2, mRNA [NM_199263] | 0.4857103 |
| TINAGL1 | ref\|Homo sapiens tubulointerstitial nephritis antigen-like 1 (TINAGL1), mRNA [NM_022164] | 2.7716244 |
| TLE2 | ref\|Homo sapiens transducin-like enhancer of split 2 (E(sp1) homolog, Drosophila) (TLE2), transcript variant 1, mRNA [NM_003260] | 0.5194139 |
| TMEM160 | ref\|Homo sapiens transmembrane protein 160 (TMEM160), mRNA [NM_017854] | 2.1245396 |
| TMEM184A | ref\|Homo sapiens transmembrane protein 184A (TMEM184A), mRNA [NM_001097620] | 1.8947364 |
| TMEM189 | ref\|Homo sapiens transmembrane protein 189 (TMEM189), transcript variant 1, mRNA [NM_199129] | 0.5426006 |
| TMEM38A | ref\|Homo sapiens transmembrane protein 38A (TMEM38A), mRNA [NM_024074] | 1.6988211 |
| TMEM99 | ref\|Homo sapiens transmembrane protein 99 (TMEM99), mRNA [NM_145274] | 0.6451725 |
| TNFSF11 | ref\|Homo sapiens tumor necrosis factor (ligand) superfamily, member 11 (TNFSF11), transcript variant 1, mRNA [NM_003701] | 1.9715826 |
| TNNI2 | ref\|Homo sapiens troponin I type 2 (skeletal, fast) (TNNI2), transcript variant 1, mRNA [NM_003282] | 1.7539933 |
| TPRA1 | ref\|Homo sapiens transmembrane protein, adipocyte asscociated 1 (TPRA1), transcript variant 3, mRNA [NM_001142646] | 0.4982772 |
| TRAF3 | ref\|Homo sapiens TNF receptor-associated factor 3 (TRAF3), transcript variant 1, mRNA [NM_145725] | 0.6110514 |
| TRIM36 | ref\|Homo sapiens tripartite motif-containing 36 (TRIM36), transcript variant 1, mRNA [NM_018700] | 0.6050752 |
| TRIM8 | ref\|Homo sapiens tripartite motif-containing 8 (TRIM8), mRNA [NM_030912] | 0.5176641 |
| TRMT1 | ref\|Homo sapiens TRM1 tRNA methyltransferase 1 homolog (S. cerevisiae) (TRMT1), transcript variant 1, mRNA [NM_017722] | 1.5800203 |
| TUSC1 | ref\|Homo sapiens tumor suppressor candidate 1 (TUSC1), mRNA [NM_001004125] | 1.5299003 |
| TUSC4 | ref\|Homo sapiens tumor suppressor candidate 4 (TUSC4), mRNA [NM_006545] | 1.5668876 |
| TYMP | ref\|Homo sapiens thymidine phosphorylase (TYMP), transcript variant 1, mRNA [NM_001113755] | 2.611361 |
| UNCX | ref\|Homo sapiens UNC homeobox (UNCX), mRNA [NM_001080461] | 2.583993 |
| VAPB | ref\|Homo sapiens VAMP (vesicle-associated membrane protein)-associated protein B and C (VAPB), mRNA [NM_004738] | 0.640583 |
| VRK1 | ref\|Homo sapiens vaccinia related kinase 1 (VRK1), mRNA [NM_003384] | 0.655908 |
| WDR31 | ref\|Homo sapiens WD repeat domain 31 (WDR31), transcript variant 1, mRNA [NM_001012361] | 0.6481941 |
| WDR51B | ref\|Homo sapiens WD repeat domain 51B (WDR51B), mRNA [NM_172240] | 0.6191338 |
| YJEFN3 | ref\|Homo sapiens YjeF N-terminal domain containing 3 (YJEFN3), nuclear gene encoding mitochondrial protein, mRNA [NM_198537] | 1.9108674 |
| ZNF205 | ref\|Homo sapiens zinc finger protein 205 (ZNF205), transcript variant 1, mRNA [NM_003456] | 1.9356601 |
| ZNF316 | ref\|PREDICTED: Homo sapiens zinc finger protein 316 (ZNF316), miscRNA [XR_078693] | 1.6822051 |
| ZNF32 | ref\|Homo sapiens zinc finger protein 32 (ZNF32), transcript variant 2, mRNA [NM_001005368] | 0.5722789 |
| ZNF467 | ref\|Homo sapiens zinc finger protein 467 (ZNF467), mRNA [NM_207336] | 1.7501889 |
| ZNF593 | ref\|Homo sapiens zinc finger protein 593 (ZNF593), mRNA [NM_015871] | 1.5814605 |
| ZNF780A | ref\|Homo sapiens zinc finger protein 780A (ZNF780A), transcript variant 2, mRNA [NM_001010880] | 2.0607938 |
| ZPLD1 | ref\|Homo sapiens zona pellucida-like domain containing 1 (ZPLD1), mRNA [NM_175056] | 1.720202 |
